# Impact of Cytochrome P450 2D6 Function on the Chiral Blood Plasma Pharmacokinetics of 3,4-Methylenedioxymethamphetamine (MDMA) and Its Phase I and II Metabolites in Humans

**DOI:** 10.1371/journal.pone.0150955

**Published:** 2016-03-11

**Authors:** Andrea E. Steuer, Corina Schmidhauser, Eva H. Tingelhoff, Yasmin Schmid, Anna Rickli, Thomas Kraemer, Matthias E. Liechti

**Affiliations:** 1 Department of Forensic Pharmacology & Toxicology, Zurich Institute of Forensic Medicine, University of Zurich, Zurich, Switzerland; 2 Psychopharmacology Research, Division of Clinical Pharmacology and Toxicology, Department of Biomedicine and Department of Clinical Research, University Hospital Basel, Basel, Switzerland; Chiba University Center for Forensic Mental Health, JAPAN

## Abstract

3,4-methylenedioxymethamphetamine (MDMA; ecstasy) metabolism is known to be stereoselective, with preference for *S*-stereoisomers. Its major metabolic step involves CYP2D6-catalyzed demethylenation to 3,4-dihydroxymethamphetamine (DHMA), followed by methylation and conjugation. Alterations in CYP2D6 genotype and/or phenotype have been associated with higher toxicity. Therefore, the impact of CYP2D6 function on the plasma pharmacokinetics of MDMA and its phase I and II metabolites was tested by comparing extensive metabolizers (EMs), intermediate metabolizers (IMs), and EMs that were pretreated with bupropion as a metabolic inhibitor in a controlled MDMA administration study. Blood plasma samples were collected from 16 healthy participants (13 EMs and three IMs) up to 24 h after MDMA administration in a double-blind, placebo-controlled, four-period, cross-over design, with subjects receiving 1 week placebo or bupropion pretreatment followed by a single placebo or MDMA (125 mg) dose. Bupropion pretreatment increased the maximum plasma concentration (C_max_) and area under the plasma concentration-time curve from 0 to 24 h (AUC_24_) of *R-*MDMA (9% and 25%, respectively) and *S-*MDMA (16% and 38%, respectively). Bupropion reduced the C_max_ and AUC_24_ of the CYP2D6-dependently formed metabolite stereoisomers of DHMA 3-sulfate, DHMA 4-sulfate, and 4-hydroxy-3-methoxymethamphetamine (HMMA sulfate and HMMA glucuronide) by approximately 40%. The changes that were observed in IMs were generally comparable to bupropion-pretreated EMs. Although changes in stereoselectivity based on CYP2D6 activity were observed, these likely have low clinical relevance. Bupropion and hydroxybupropion stereoisomer pharmacokinetics were unaltered by MDMA co-administration. The present data might aid further interpretations of toxicity based on CYP2D6-dependent MDMA metabolism.

## Introduction

3,4-Methylenedioxymethamphetamine (MDMA; ecstasy) is an illicit amphetamine derivative that is used recreationally, usually as a 1:1 mixture of its *R*- and *S*-enantiomers. MDMA enhances feelings of energy, friendliness, euphoria, and empathy [1, 2]. In animals, the *S*-enantiomer of MDMA or its active metabolite 3,4-methylenedioxyamphetamine (MDA) are more potent than the *R*-enantiomers [3–6]. Addionally, *S*-MDMA has more amphetamine distinctive effects [7] whereas *R*-MDMA and *R*-MDA have more hallucinogen-like effects than the *S*-enantiomers [8] Acute toxicity (e.g., tachycardia, hypertension, hyperthermia, and hepatotoxicity) and even fatalities have been associated with the short-term use of MDMA [9, 10]. Neurotoxic effects on serotonergic neurons, presumably via the bioactivation and formation of glutathione adducts [11–18], are still being investigated and controversially discussed in terms of species-dependence and dosing [19–21]. Importantly, alterations of cytochrome P450 (CYP)-mediated MDMA metabolism were shown to influence neurotoxicity [11, 17, 22].

**Fig 1:**
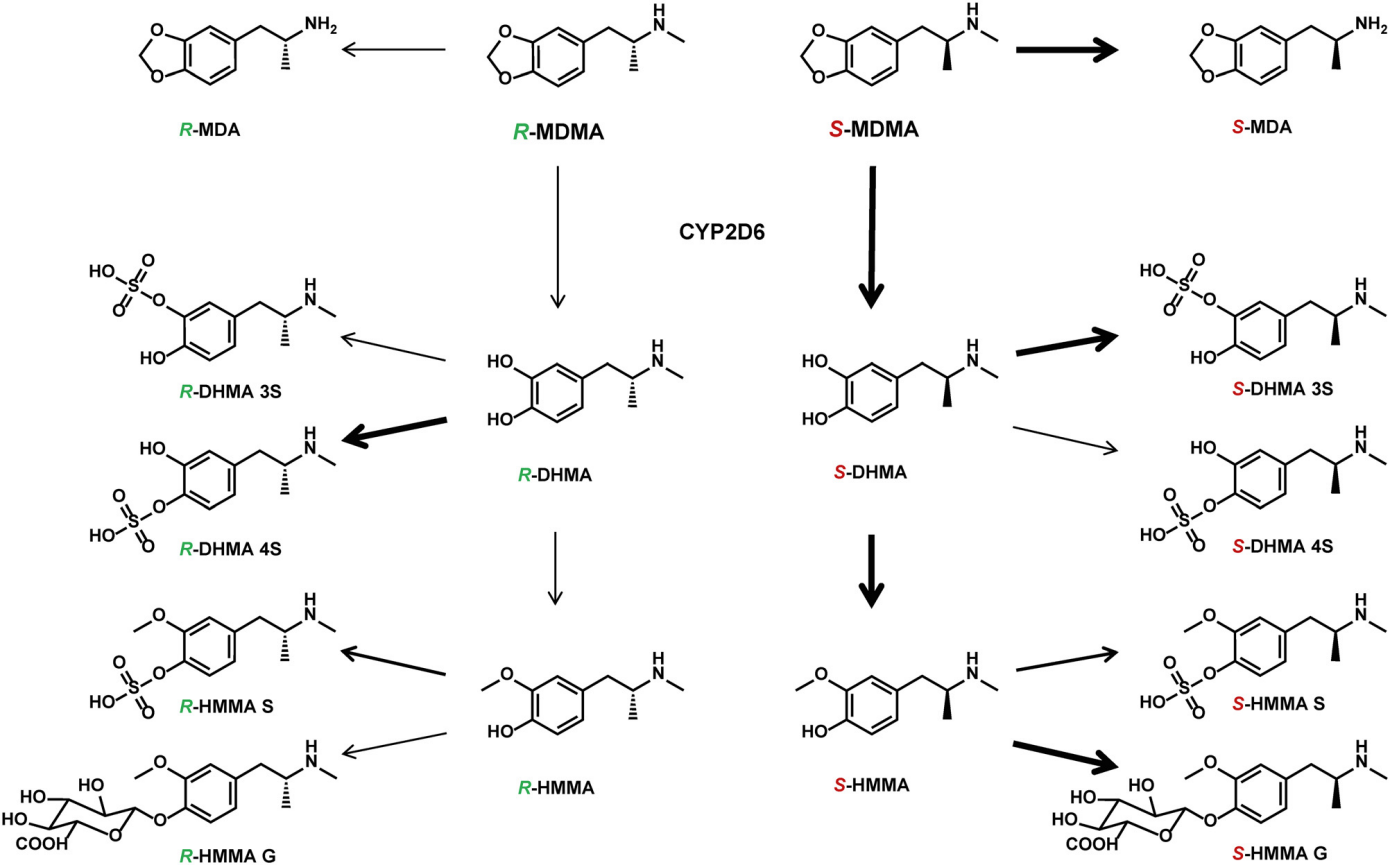
Main metabolic steps of MDMA. MDMA is mainly metabolized by CYP2D6-mediated *O*-demethylenation to 3,4-dihydroxymethamphetamine (DHMA), followed by catechol-*O*-methyltransferase (COMT) *O*-methylation mainly to 4-hydroxy-3-methoxymethamphetamine (HMMA). DHMA is further sulfated mainly by sulfotransferases (SULTs) to DHMA 3-sulfate (DHMA 3S) and DHMA 4-sulfate (DHMA 4S). HMMA can be further conjugated by UDP-glucuronyltransferases to HMMA glucuronide (HMMA G) or sulfotransferases to HMMA sulfate (HMMA S). Bold arrows indicate preferences in depicted metabolic reaction for *R*- or *S*-stereoisomer, respectively. Only sulfation of HMMA revealed no enantiomeric preferences.

As shown in Fig 1, in humans, MDMA is mainly metabolized by CYP2D6-mediated *O*-demethylenation to 3,4-dihydroxymethamphetamine (DHMA), followed by catechol-*O*-methyltransferase (COMT) *O*-methylation mainly to 4-hydroxy-3-methoxymethamphetamine (HMMA). DHMA is further sulfated mainly by sulfotransferases (SULTs) to DHMA 3-sulfate and DHMA 4-sulfate. HMMA can be further conjugated by UDP-glucuronyltransferases (UGTs) or SULTs. DHMA 3-sulfate, HMMA sulfate, and HMMA glucuronide were shown to be the main metabolites in plasma [23] and urine [24], whereas free DHMA and HMMA were not detectable or only in negligible amounts. A minor pathway includes demethylation to MDA, mainly by CYP2B6, CYP1A2, and CYP3A4 [25, 26], followed by demethylenation, *O*-methylation, and conjugation [27–29]. Differences in the metabolism and plasma pharmacokinetics of the two enantiomers of MDMA were reported *in vitro* [26, 30–32] and *in vivo* [9, 23, 33–36], with higher *R*-MDMA blood concentrations and preferred elimination (metabolism and excretion) of *S*-stereoisomers.

**Fig 2:**
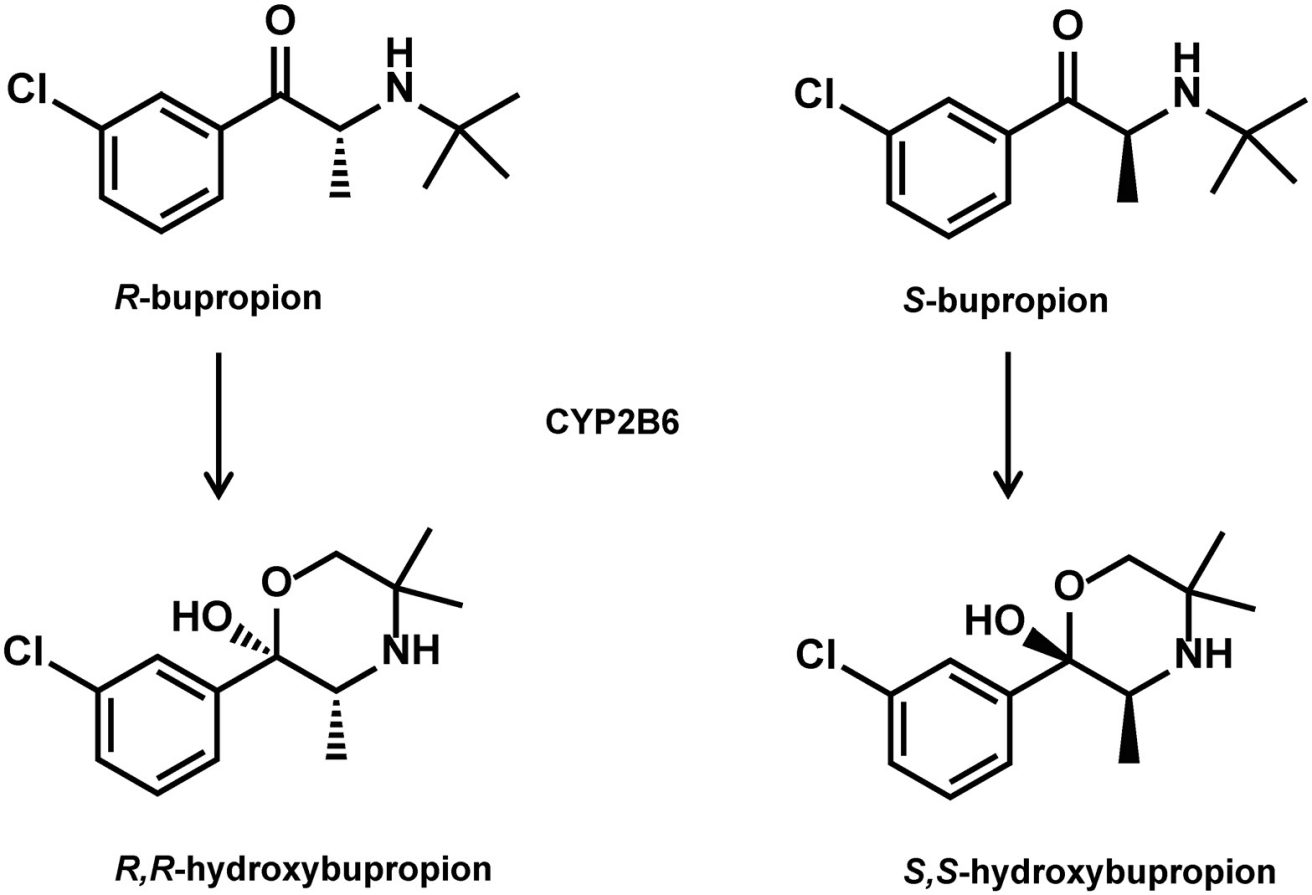
Main metabolic step of bupropion. Chemical structures of bupropion enantiomers and their main metabolites R,R- and S,S-hydroxybupropion formed through CYP2B6-mediated hydroxylation.

Although CYP2D6 only accounts for approximately 2% of hepatic CYP enzymes, it is responsible for approximately 19% of drug metabolism [37, 38]. Among the CYP enzymes, CYP2D6 is the most susceptible to genetic polymorphisms, with more than 100 allelic variants [37, 38], which can be phenotypically classified into four main groups: poor metabolizer (PM), intermediate metabolizer (IM), extensive metabolizer (EM), and ultra-rapid metabolizer (UM). Extensive metabolizers have fully functional enzymes, whereas IMs and PMs exhibit reduced activity and UMs exhibit increased enzyme activity. Among Caucasians, EMs, IMs, PMs, and UMs represent approximately 70–80%, 10–17%, 5–10%, and 3–5% of the population, respectively. The participation of CYP2D6 in the metabolism of MDMA may suggest that individuals with extensive or even ultrafast metabolism might be at a higher risk for neurotoxic effects, whereas IMs and/or PMs are more prone to acute MDMA toxicity [17]. However, finding sufficient numbers of UMs and/or PMs for controlled MDMA administration studies is rather difficult. Co-administering known CYP2D6 inhibitors (e.g., the selective serotonin reuptake inhibitor [SSRI] paroxetine or fluoxetine) might mimic the IM or PM genotype. Segura et al. successfully used this approach with paroxetine as a CYP2D6 inhibitor in seven EMs who were orally administered MDMA [39]. However, the analysis was only performed with racemic MDMA and free metabolites after conjugate cleavage and did not take into account potential differences in stereoisomers and the abundance of all metabolites. Bupropion acts as a selective norepinephrine/dopamine reuptake inhibitor. It was recently used in an interaction study with MDMA to explore the role of dopamine in the psychotropic effects of MDMA [40]. Bupropion is commonly used for the treatment of depression and smoking cessation and has been studied for a number of other diseases (e.g., bipolar disorder and attention-deficit/hyperactivity disorder) and weight loss [41, 42]. It is extensively metabolized mainly to hydroxybupropion, primarily by CYP2B6 as shown in Fig 2, and to a lesser extent to dehydrobupropion (erythrohydro- and threohydrobupropion). Plasma pharmacokinetics were shown to differ between stereoisomers with higher concentrations of *R*-bupropion and *R*,*R*-hydroxybupropion [43], whereas higher pharmacological potency of *S*,*S*-hydroxybupropion was found compared with both *R*,*R*-hydroxybupropion and racemic bupropion. The effects were comparable between racemic bupropion and each of its enantiomers *in vitro*, most likely because of its rapid racemization under physiological conditions. Bupropion and particularly erythrohydro- and threohydrobupropion have previously been shown to inhibit CYP2D6 [41, 44, 45].

The aim of the present study was to assess the contribution of CYP2D6 to the chiral pharmacokinetics of MDMA and its phase I and II metabolites in samples that were collected during a previous pharmacodynamic interaction study [40]. Thirteen EMs and three IMs participated in the study, allowing us to explore the effects of genetic differences in CYP2D6 function on MDMA metabolism. Bupropion was also repeatedly administered prior to MDMA administration to inhibit CYP2D6 activity, thus allowing exploration of the effect of pharmacologically inhibiting CYP2D6 on MDMA metabolism in all of the study participants.

## Materials and Methods

### Chemicals and reagents

The sources of the chemicals and reagents that were used for the MDMA analysis are provided in detail in a previous study [46]. Methanolic solutions of hydrochlorides of racemic bupropion (1 mg/ml) and bupropion-d9 (0.1 mg/ml) and acetonitrilic solutions of hydroxybupropion (1 mg/ml) and hydroxybupropion-d6 (0.1 mg/ml) were obtained from Cerilliant and delivered via Sigma-Aldrich (Buchs, Switzerland).

### Clinical study

The study used a double-blind, placebo-controlled, crossover design with four experimental test sessions (placebo+placebo, bupropion+placebo, placebo+MDMA, and bupropion+MDMA) in 16 healthy Caucasian subjects (eight men and eight women) with a mean ± SD age of 24.3 ± 2.2 years and a body mass index of 22.7 ± 2.1 kg/m^2^ as described in detail in a previous study [40]. Washout periods between test sessions were at least 10 days. The study was conducted in accordance with the Declaration of Helsinki and approved by the Ethics Committee of the Canton of Basel, Switzerland, and the Swiss Agency for Therapeutic Products (Swissmedic). The study was registered at ClinicalTrials.gov (NCT01771874). All of the subjects provided written informed consent and were paid for their participation. Bupropion (Wellbutrin XR 150 mg, GlaxoSmithKline, Munchenbuchsee, Switzerland) or placebo was administered once daily at a dose of 150 mg for 3 days, followed by administration of 300 mg once daily for 4 days before the test days. On the test day, the last dose of bupropion or placebo (300 mg) was administered at 8:00 AM, 2 h before MDMA administration (125 mg MDMA hydrochloride, Lipomed, Arlesheim, Switzerland), or placebo was administered at 10:00 AM. Blood samples were collected -2, 0, 0.33, 0.67, 1, 1.5, 2, 2.5, 3, 4, 6, 8, and 24 h after MDMA or placebo administration. All of the subjects were genotyped [47] and phenotyped [48] for CYP2D6 activity. The study included 13 EMs, three IMs, and no PMs (genotyping and phenotyping congruent) [40]. Subjects were also genotyped for CYP2B6 (reduced-activity single nucleotid polymorphism rs3745274 (516G>T, CYP2B6*6 or CYP2B6*9) using commercial TaqMan assay (LuBio Science, Lucerne, Switzerland). There were 9 subjects with the G/G, 6 subjects with the G/T and one subject with the T/T genotype. Subjects with G/T or T/T genotype were considered to have a reduced CYP2B6 function.

### Chiral analysis of MDMA and metabolites

Blood plasma samples were analyzed non-stereoselectively as reported previously [40] and reanalyzed for the present report using liquid chromatography-mass spectrometry/mass spectrometry after chiral derivatization with Marfey’s reagent as described previously [46]. The method was fully validated, including selectivity, recovery, matrix effects, bias and imprecision, stability, and limit of quantification [46].

### Chiral analysis of bupropion and hydroxybupropion

Blood plasma samples were analyzed stereoselectively for *R-* and *S-*bupropion and their major metabolites *R*,*R-* and *S*,*S-*hydroxybupropion according to a previous study [43] with slight modifications as described in the supporting information (S1 text). The method was fully validated according to national and international guidelines [49, 50].

### Pharmacokinetic analysis

Maximum plasma concentration (C_max_), time to reach C_max_ (t_max_), area under the plasma concentration-time curve from 0 to 24 h (AUC_24_), AUC from time 0 to infinity (AUC_∞_), and elimination half-life (t_1/2_) were calculated for all analytes and apparent total clearance (Cl/F) for *R*- and *S*-MDMA using noncompartmental methods (PK solutions 2.0 software, Summit Research Services, Montrose, CO, USA). The time interval after dosing until detection of the first positive sample was designated as the time of first detection (t_onset_). Wilcoxon matched-pairs tests (95% confidence interval) were used to test for within-subjects differences in pharmacokinetic parameters between the *R* and *S* stereoisomers and between different treatment conditions (placebo-MDMA *vs*. bupropion-MDMA, bupropion-placebo *vs*. bupropion-MDMA). Mann-Whitney tests (95% confidence interval) were used to test for between-subject differences between CYP2D6 EMs (*n* = 13) and CYP2D6 IMs (*n* = 3). The statistical analyses were performed using Prism 6.00 software (GraphPad Software, La Jolla, CA, USA).

## Results

### Chiral pharmacokinetics of MDMA and metabolites

**Fig 3:**
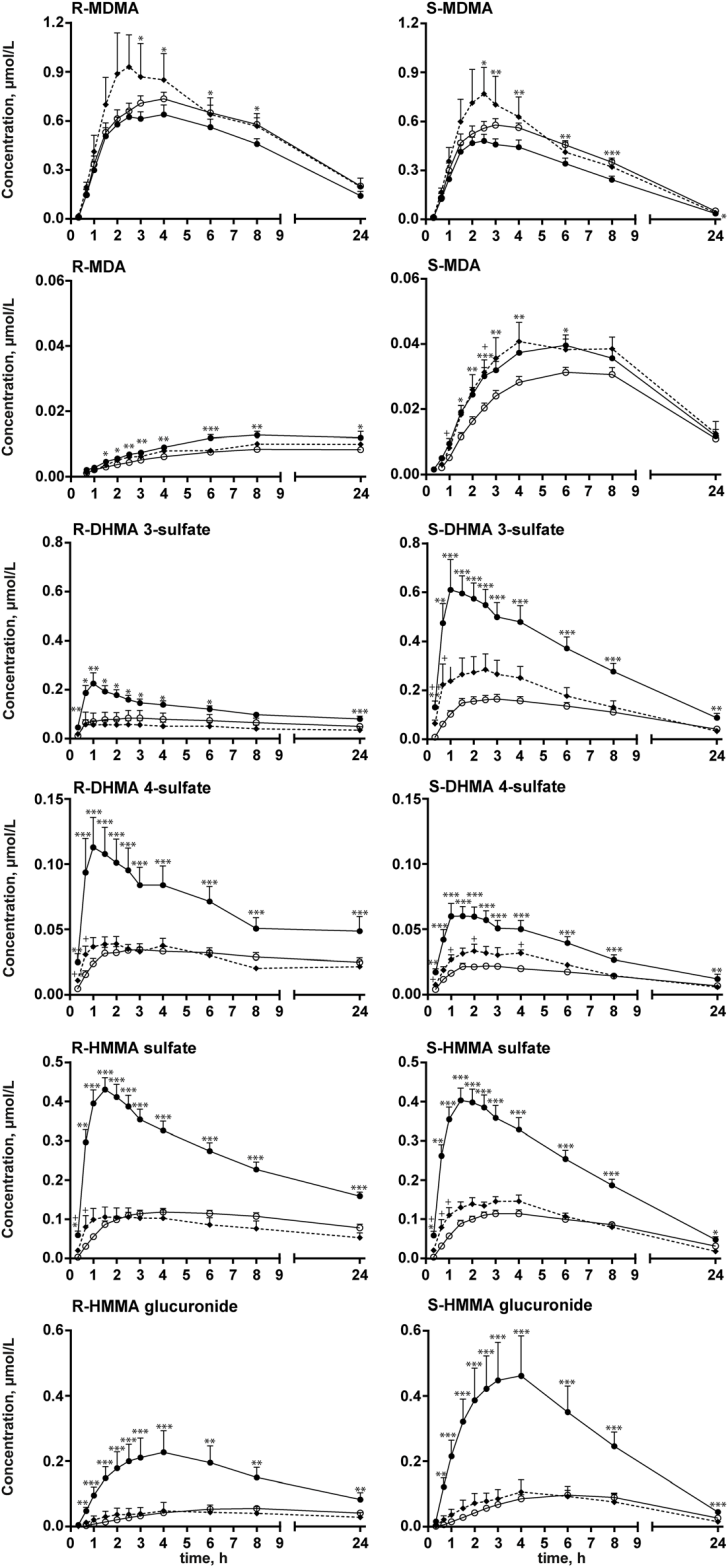
Plasma-concentration time profiles of MDMA and metabolites. Plasma-concentration time profiles for *R-* and *S-*MDMA and *R-* and *S-*MDA, *R-* and *S-*DHMA 3-sulfate and *R-* and *S-*DHMA 4-sulfate, and *R-* and *S-*HMMA sulfate and *R-* and *S-*HMMA glucuronide. Black circles and solid lines represent 13 CYP2D6 extensive metabolizers (EM) after placebo-MDMA administration. Triangles and dotted lines represent three CYP2D6 intermediate metabolizers (IM) after placebo+MDMA administration. Open circles and solid lines represent 13 CYP2D6 EMs after bupropion+MDMA administration. The data are expressed as mean and SEM. *p < 0.05, **p < 0.01, ***p < 0.001, significant within-subject difference between placebo+MDMA and bupropion+MDMA in CYP2D6 EM subjects (Wilcoxon matched-pairs test); ^+^*p* < 0.05, ^++^*p* < 0.01, significant difference between placebo+MDMA-treated CYP2D6 IMs and bupropion+MDMA-treated CYP2D6 EMs (Mann-Whitney test).

Plasma concentration-time profiles for MDMA and metabolites in placebo+MDMA-treated CYP2D6 EMs and CYP2D6 IMs and bupropion+MDMA-treated EMs are presented in Fig 3. The pharmacokinetic parameters for all three groups are listed in Table 1. MDMA concentrations were significantly higher in bupropion+MDMA CYP2D6 EMs compared with placebo+MDMA-treated CYP2D6 EMs (9% and 16% increases in C_max_ and 25% and 38% increases in AUC_24_ for *R*- and *S*-enantiomers, respectively). MDMA concentrations marginally increased in CYP2D6 IMs compared with the other groups, but the difference was not significant because of the small number of subjects in the IM group. Bupropion pretreatment reduced the elimination of MDMA, with a significant prolongation of t_1/2_ for *R*-MDMA (31%) and *S*-MDMA (26%) and a reduction of Cl/F (-23% and -31%, respectively). The elimination of MDMA was also marginally reduced in CYP2D6 IMs compared with EMs, but the reduction was not significant.

**Table 1:** Pharmacokinetic data of MDMA.

	Cmax [μmol/l]			tmax [h]		AUC0-24h [μmol/l*h-1]			AUCtotal [μmol/l*h-1]		t_1/2_ [h]		CL/F [l/h]	
	*R*	*S*	*R/S*	*R*	*S*	*R*	*S*	*R/S*	*R*	*S*	*R*	*S*	*R*	*S*
MDMA														
EM	0.69 (0.17)	0.56 (0.15)	1.2 (0.1)	2.9 (0.8)	2.1 (0.7)	8.7 (2.3)	4.9 (1.4)	1.8 (0.2)	10.4 (3.6)	5.1 (1.5)	8.2 (2.4)	4.5 (0.6)	40.4 (14.2)	72.8 (26.2)
IM	0.85 (0.30)	0.72 (0.23)	1.2 (0.1)	2.8 (0.9)	2.3 (0.6)	11.4 (3.3)	6.8 (2.0)	1.7 (0.1)	14.5 (4.3)	7.1 (2.0)	10.3 (3.6)	5.1 (0.9)	29.8 (7.4)	50.4 (12.6)
EM/Bup	0.75 (0.16)	0.65 (0.15)	1.1 (0.1)	3.3 (1.0)	2.5 (0.6)	10.9 (2.4)	6.8 (1.5)	1.6 (0.1)	14.2 (3.7)	7.2 (1.7)	10.8 (1.9)	5.7 (0.5)	31.3 (8.2)	50.5 (13.5)
MDA														
EM	0.014 (0.006)	0.041 (0.012)	0.3 (0.1)	12.5 (8.1)	4.6 (0.8)	0.26 (0.10)	0.61 (0.19)	0.4 (0.1)	n.d.	0.76 (0.27)	n.d.	9.2 (2.3)		
IM	0.011 (0.002)	0.042 (0.006)	0.3 (0.0)	18.7 (9.2)	4.4 (0.5)	0.21 (0.050)	0.65 (0.15)	0.3 (0.0)	n.d.	0.87 (0.31)	n.d.	10.6 (3.3)		
EM/Bup	0.010 (0.005)	0.031 (0.007)	0.3 (0.1)	14.9 (8.8)	5.2 (0.8)	0.19 (0.076)	0.49 (0.14)	0.4 (0.1)	n.d.	0.69 (0.17)	n.d.	11.1 (1.8)		
DHMA 3S														
EM	0.15 (0.080)	0.59 (0.25)	0.3 (0.1)	2.8 (1.8)	1.5 (0.3)	2.4 (1.0)	6.3 (2.7)	0.4 (0.1)	4.7 (2.5)	7.1 (3.1)	20.2 (6.2)	7.3 (1.5)		
IM	0.055 (0.030)	0.28 (0.11)	0.2 (0.0)	1.7 (0.9)	1.9 (0.5)	0.98 (0.64)	3.0 (1.0)	0.3 (0.1)	2.6 (2.4)	3.3 (1.1)	27.4 (11.5)	7.0 (0.4)		
EM/Bup	0.050 (0.025)	0.20 (0.088)	0.3 (0.1)	3.8 (1.1)	2.4 (0.6)	0.96 (0.44)	2.8 (1.3)	0.4 (0.1)	3.1 (2.1)	3.7 (2.1)	46.2 (32.5)	11.3 (2.8)		
DHMA 4S														
EM	0.080 (0.048)	0.055 (0.025)	1.5 (0.5)	2.0 (0.8)	1.7 (0.5)	1.3 (0.74)	0.64 (0.27)	2.0 (0.7)	2.4 (1.7)	0.80 (0.37)	17.5 (5.5)	8.1 (1.2)		
IM	0.035 (0.005)	0.029 (0.005)	1.2 (0.1)	3.0 (1.5)	2.1 (0.5)	0.54 (0.14)	0.40 (0.11)	1.5 (0.6)	1.5 (1.0)	0.59 (0.30)	18.7 (15.6)	12.1 (5.0)		
EM/Bup	0.037 (0.015)	0.021 (0.007)	1.7 (0.6)	3.2 (1.1)	2.4 (0.9)	0.71 (0.30)	0.32 (0.11)	2.1 (0.7)	2.9 (2.2)	0.48 (0.19)	49.9 (29.3)	14.3 (3.6)		
HMMA S														
EM	0.37 (0.11)	0.41 (0.13)	0.9 (0.2)	2.0 (1.3)	1.6 (0.5)	5.5 (1.4)	4.2 (1.2)	1.3 (0.2)	9.6 (2.7)	4.6 (1.2)	17.5 (6.6)	6.6 (0.80)		
IM	0.10 (0.041)	0.14 (0.022)	0.7 (0.2)	1.7 (0.8)	2.5 (0.3)	1.7 (0.70)	1.7 (0.30)	1.0 (0.3)	3.4 (1.3)	1.9 (0.34)	21.2 (0.40)	6.9 (0.50)		
EM/Bup	0.14 (0.039)	0.11 (0.033)	1.1 (0.2)	4.2 (1.1)	2.8 (0.6)	2.1 (0.67)	1.6 (0.51)	1.3 (0.4)	7.1 (4.6)	2.2 (0.75)	34.4 (23.9)	11.1 (2.5)		
HMMA G														
EM	0.21 (0.19)	0.49 (0.39)	0.5 (0.2)	3.7 (2.1)	2.9 (1.0)	3.3 (2.1)	5.2 (3.7)	0.6 (0.2)	5.5 (4.3)	5.5 (3.9)	16.0 (7.9)	6.1 (1.2)		
IM	0.043 (0.038)	0.099 (0.060)	0.4 (0.1)	4.8 (0.5)	4.0 (0.1)	0.84 (0.74)	1.3 (0.77)	0.6 (0.2)	2.0 (1.7)	1.5 (0.83)	29.3 (12.4)	7.1 (0.4)		
EM/Bup	0.049 (0.027)	0.088 (0.044)	0.6 (0.1)	7.6 (1.2)	4.9 (1.0)	0.96 (0.54)	1.3 (0.78)	0.7 (0.1)	2.3 (1.5)	1.7 (1.0)	31.7 (16.8)	9.0 (2.8)		

For MDA, C_max_ (-27% for *R*-MDA, -24% for *S*-MDA) and AUC_24_ values (-26% for *R*-MDA, -20% for *S*-MDA) were reduced by pretreatment with bupropion. Although *R*-MDA in CYP2D6 IMs showed the same effect, *S*-MDA in CYP2D6 IMs was within the same range as in CYP2D6 EMs. The t_max_ and t_1/2_ values for *S*-MDA were not significantly different. The t_1/2_ for *R*-MDA could not be calculated because of the limited number of data points in the elimination phase.

The concentrations of all phase II metabolites significantly decreased after bupropion pretreatment compared with placebo pretreatment, with lower C_max_ and AUC_24_ values for *R*-DHMA 3-sulfate (-68% and -61%), *S*-DHMA 3-sulfate (-67% and -56%), *R*-DHMA 4-sulfate (-54% and -43%), *S*-DHMA 4-sulfate (-61% and -50%), *R*-HMMA sulfate (-62% and -62%), *S*-HMMA sulfate (-72% and -61%), *R*-HMMA glucuronide (-77% and -71%), and *S*-HMMA glucuronide (-82% and -74%), respectively. CYP2D6 IMs presented no significant differences compared with bupropion-treated EMs, although for *S*-enantiomers sulfates co-administration of bupropion apparently elevated C_max_ while AUC_24_ remained unaltered. The t_max_ was reached significantly later for all phase II metabolites, varying between 40% and 100% for *R*-stereoisomers and between 45% and 70% for *S*-stereoisomers, and t_1/2_ was prolonged up to 200% for *R*-stereoisomers and up to 70% for *S*-stereoisomers after bupropion pretreatment compared with placebo pretreatment. Similar changes were observed in CYP2D6 IMs compared with EMs, although to a much lesser extent, especially for *R*-stereoisomers.

The t_onset_ was comparable between CYP2D6 EMs and IMs for all of the analytes. With bupropion pretreatment, the first detection of *R*-HMMA sulfate, *S*-HMMA sulfate, *R*-HMMA glucuronide, and *S*-HMMA glucuronide was significantly delayed (mean placebo-pretreated CYP2D6 EMs *vs*. bupropion-pretreated CYP2D6 EMs: 0.33 h *vs*. 0.53 h, 0.33 h *vs*. 0.56 h, 0.41 h *vs*. 0.73 h, and 0.38 h *vs*. 0.71 h, respectively).

**Fig 4:**
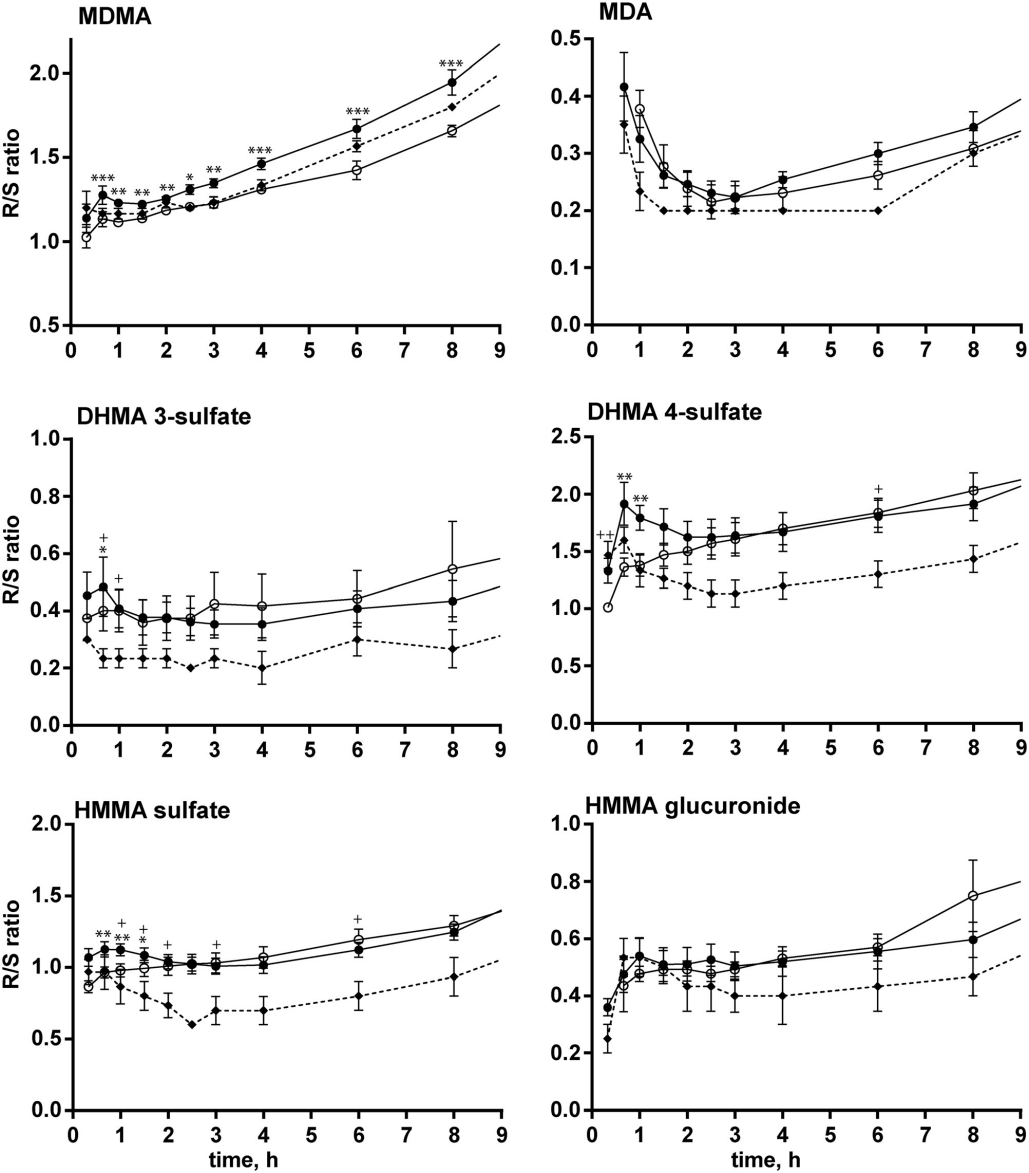
*R/S* ratios of MDMA and metabolites. *R/S* concentration ratios for MDMA, MDA, DHMA 3-sulfate, DHMA 4-sulfate, HMMA sulfate, and HMMA glucuronide over time. Black circles and solid lines represent 13 CYP2D6 extensive metabolizers (EM). Triangles and dotted lines represent three CYP2D6 intermediate metabolizers (IM). Open circles and solid lines represent 13 CYP2D6 EMs after pretreatment with bupropion. The data are expressed as mean and SEM. *p < 0.05, **p < 0.01, ***p < 0.001, significant within-subject difference between placebo+MDMA and bupropion+MDMA in CYP2D6 EM subjects (Wilcoxon matched-pairs test); ^+^*p* < 0.05, ^++^*p* < 0.01, significant differences between placebo+MDMA-treated CYP2D6 IMs and bupropion+MDMA-treated CYP2D6 EMs (Mann-Whitney test).

Generally, alterations in CYP2D6 metabolism that were induced by bupropion pretreatment influenced both stereoisomers but to slightly different extents. The *R/S* concentration ratios over time for MDMA and all metabolites for the different treatment groups are shown in Fig 4. The MDMA *R/S* ratio was significantly lower after pretreatment with bupropion (-6% for C_max_, -9% for AUC_24_), as was the MDA *R/S* ratio (-19% for C_max_, -17% for AUC_24_). The HMMA sulfate *R/S* ratio was significantly higher (+21% for C_max_). Although CYP2D6 IMs appeared to have slightly lower mean *R/S* ratios than bupropion-pretreated EMs (mainly for sulfate conjugates), the trend was not significant with regard to C_max_ and AUC_24_ values.

**Fig 5:**
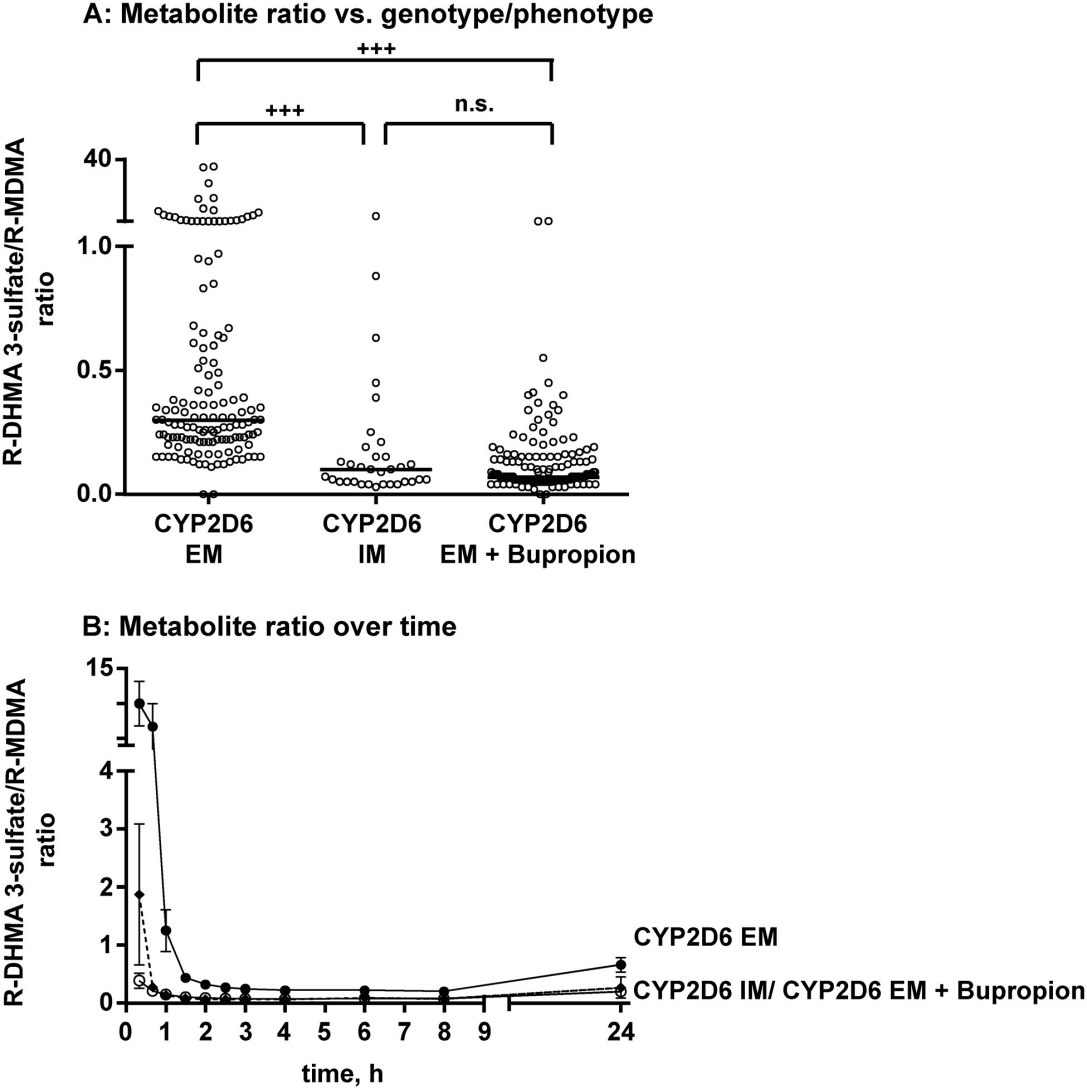
Metabolite ratios. (a) Metabolite ratio of *R*-DHMA 3-sulfate to *R*-MDMA in placebo-pretreated CYP2D6 extensive metabolizers (EM), intermediate metabolizers (IM), and EMs after pretreatment with bupropion. The data represent single measurements of 13 participants (placebo-pretreated EMs and bupropion-pretreated EMs) or three IMs at different time points after MDMA administration (0.33–24 h). ^+++^*p* < 0.001, significant difference between placebo+MDMA-treated CYP2D6 IMs and bupropion+MDMA-treated CYP2D6 EMs (Mann-Whitney test). (b) Metabolite ratio of *R*-DHMA 3-sulfate to *R*-MDMA in CYP2D6 EMs, IMs, and EMs after pretreatment with bupropion over time. Black circles and solid lines represent extensive metabolizers (EM). Triangles and dotted lines represent intermediate metabolizers (IM). Open circles and solid lines represent EMs after pretreatment with bupropion. The data are expressed as mean and SEM.

Metabolite/MDMA ratios at each time point for CYP2D6 EMs, IMs, and bupropion-pretreated EMs are shown in Fig 5a, exemplified by the *R*-DHMA 3-sulfate/*R*-MDMA ratio. Significant differences were observed between CYP2D6 EMs and IMs and between placebo-pretreated EMs and bupropion-pretreated EMs, whereas IMs and bupropion-pretreated EMs did not differ significantly. The same was observed for all of the other metabolites. Despite significant differences between means, the ranges considerably overlapped. Fig 5b shows the changes in metabolite/MDMA ratios over time, with the highest values (mean: 9.9 in placebo-pretreated EMs, 1.9 in IMs, and 0.4 in bupropion-pretreated EMs) in the first hour after MDMA administration, decreasing to rather constant values after approximately 2 h (mean: 0.22 in placebo-pretreated EMs, 0.10 in IMs, 0.10 in bupropion-pretreated EMs). The initial high ratios were not observed after bupropion-pretreatment. Similar changes in metabolite/MDMA ratios were observed for all of the other metabolites, with the following mean initial and constant ratios, respectively: *S*-DHMA 3-sulfate (33 and 1.1 in placebo-pretreated EMs, 8.8 and 0.42 in IMs, and 1.8 and 0.34 in bupropion-pretreated EMs), *R*-DHMA 4-sulfate (4.9 and 0.13 in placebo-pretreated EMs, 0.94 and 0.05 in IMs, and 0.89 and 0.05 in bupropion-pretreated EMs), *S*-DHMA 4-sulfate (3.8 and 0.11 in placebo-pretreated EMs, 0.91 and 0.05 in IMs, and 0.60 and 0.04 in bupropion-pretreated EMs), *R*-HMMA sulfate (15.8 and 0.54 in placebo-pretreated EMs, 1.9 and 0.13 in IMs, and 0.31 and 0.13 in bupropion-pretreated EMs), *S*-HMMA sulfate (17.7 and 0.86 in placebo-pretreated EMs, 2.3 and 0.28 in IMs, and 0.26 and 0.21 in bupropion-pretreated EMs), *R*-HMMA glucuronide (1.3 and 0.40 in placebo-pretreated EMs, 0.05 and 0.05 in IMs, and 0.09 and 0.05 in bupropion-pretreated EMs), and *S*-HMMA glucuronide (5.6 and 1.3 in placebo-pretreated EMs, 0.30 and 0.20 in IMs, and 0.10 and 0.20 in bupropion-pretreated EMs).

**Fig 6:**
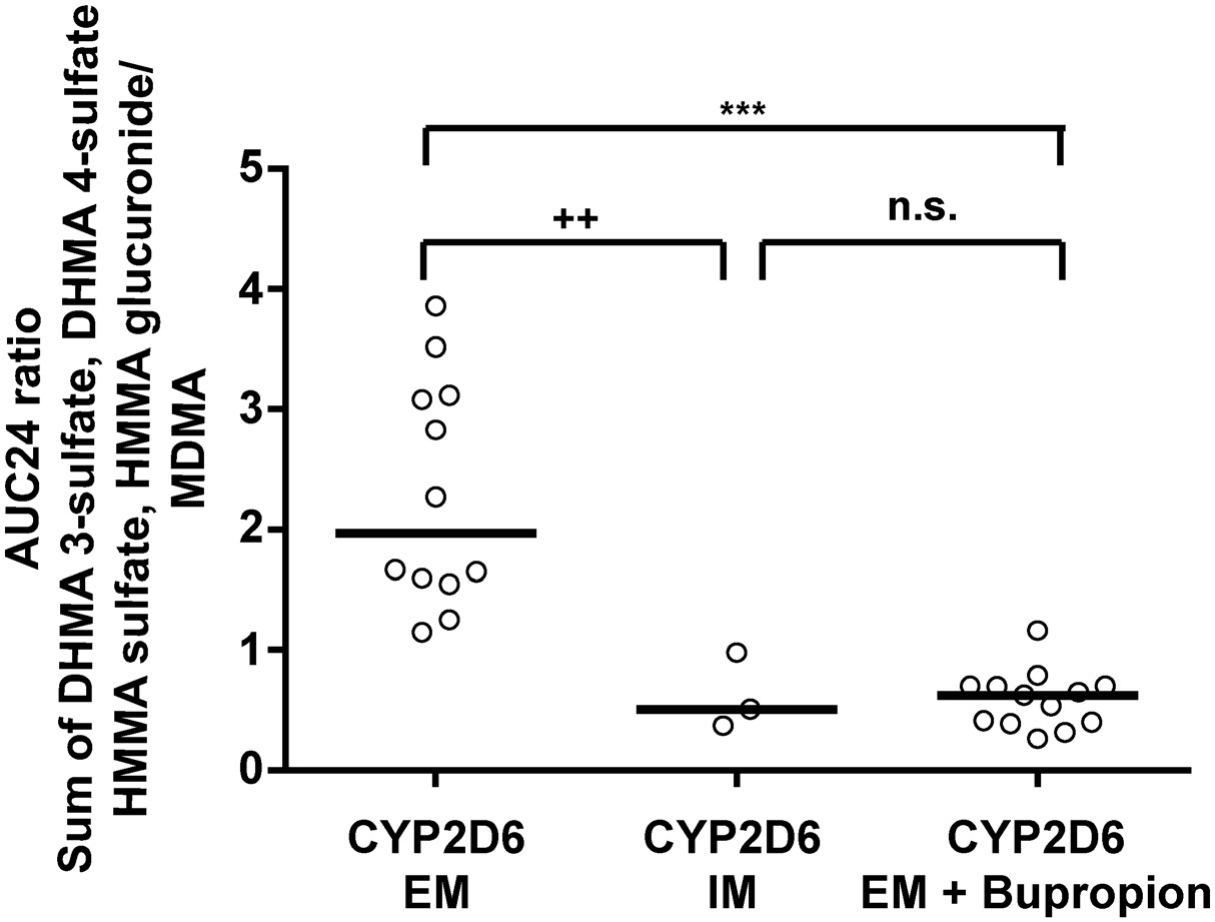
Influence of CYP2D6 genotype on MDMA metabolite formation. Correlations between CYP2D6 genotype (extensive metabolizers [EM], intermediate metabolizers [IM], and bupropion-pretreated EMs) and AUC_24_ ratio of the sum of CYP2D6-dependently formed metabolites (DHMA 3-sulfate, DHMA 4-sulfate, HMMA sulfate, and HMMA glucuronide) to MDMA. The data represent individual participants (EM, *n* = 13; IM, *n* = 3; EMs after bupropion pretreatment, *n* = 13). *p < 0.05, **p <0.01, ***p < 0.001, significant difference between EMs and bupropion-pretreated EMs (Wilcoxon matched-pairs test); ^+^*p* < 0.05, ^++^*p* < 0.01, significant difference between bupropion-pretreated EMs and IMs (Mann-Whitney test).

The AUC_24_ metabolite ratios of all CYP2D6-mediated metabolites (*R*,*S*-DHMA 3-sulfate, *R*,*S*-DHMA 4-sulfate, *R*,*S*-HMMA sulfate, and *R*,*S-*HMMA glucuronide) to *R/S*-MDMA were significantly different between placebo-pretreated CYP2D6 EMs and IMs and between placebo-pretreated EMs and bupropion-pretreated EMs but not between IMs and bupropion-pretreated EMs (Fig 6). The same result was found for the AUC_24_ ratio between each single stereoisomer and MDMA.

### Chiral pharmacokinetic analysis of bupropion and hydroxybupropion

Chiral separation was achieved on a chiral AGP column with sufficient separation of each stereoisomer. The respective chromatogram is provided in the Supporting Information (S1 Fig). The results for recovery, matrix effects, bias, and imprecision are provided in the Supporting Information (S1 Table). The method was sensitive and selective and showed a linear correlation for each bupropion enantiomer (from 0.5 to 200 ng/ml) and for the two hydroxybupropion stereoisomers (from 2.5 to 1000 ng/ml).

**Fig 7:**
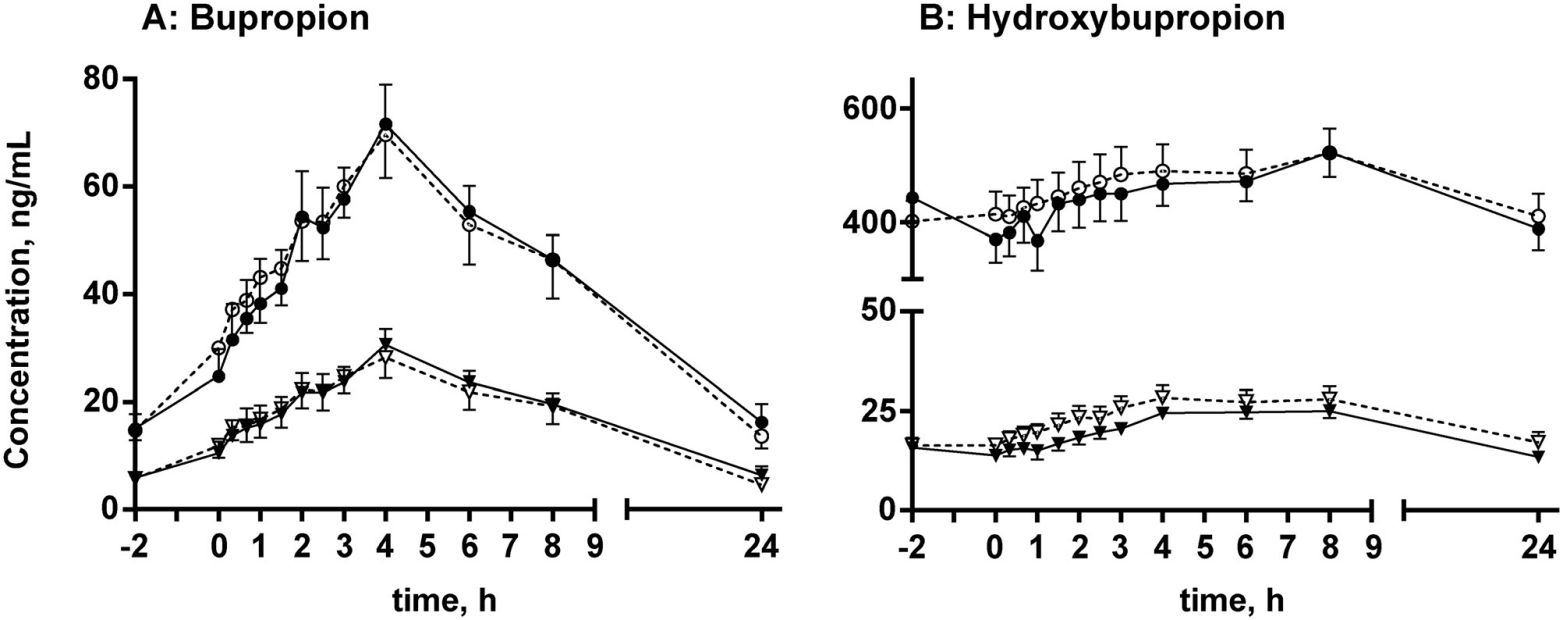
Chiral plasma-concentration time profiles for bupropion and hydroxybupropion. Plasma-concentration time profiles for *R*- and *S*-bupropion (A) and *R*,*R*- and *S*,*S-*hydroxybupropion (B) after co-administration of placebo (black) or MDMA (white). Circles represent *R*- and *R*,*R*-stereoisomers. Triangles represent *S*- and *S*,*S*-stereoisomers. The data are expressed as mean and SEM (*n* = 16 participants). Differences between placebo and MDMA were not statistically significant.

Mean plasma concentration-time profiles for *R*- and *S*-bupropion and *R*,*R*- and *S*,*S*-hydroxybupropion after placebo and MDMA co-administration are shown in Fig 7, respectively. The calculated pharmacokinetic parameters are listed in Table 2. MDMA co-administration did not significantly alter the concentrations of either *R*- and *S*-bupropion or *R*,*R*- and *S*,*S*-hydroxybupropion.

**Table 2:** Pharmacokinetic data of Bupropion (P placebo, M MDMA).

	Genotype CYP2B6	C_max_ [ng/ml]		t_max_ [h]		AUC0-24h [ng/ml*h-1]		AUCtotal [ng/ml*h-1]		t1/2 [h]	
		*R/ R*,*R*	*S/ S*,*S*	*R/ R*,*R*	*S/ S*,*S*	*R/ R*,*R*	*S/ S*,*S*	*R/ R*,*R*	*S/ S*,*S*	*R/ R*,*R*	*S/ S*,*S*
Bupropion											
P	normal	51.2 (6.0)	22.8 (12.3)	5.0 (2.1)	5.0 (1.4)	807 (387)	346 (172)	942 (444)	390 (186)	8.4 (1.8)	7.3 (1.6)
P	reduced	68.0 (15.6)	28.6 (3.8)	5.8 (2.5)	5.2 (0.6)	1099 (112)	442 (52.6)	1334 (253)	518 (110)	8.9 (2.9)	7.8 (2.8)
M	normal	53.5 (19.4)	21.9 (6.7)	5.2 (2.6)	6.2 (2.4)	822 (237)	344 (75.1)	969 (284)	395 (109)	8.5 (3.3)	7.5 (2.7)
M	reduced	68.7 (23.6)	31.4 (17.0)	5.6 (2.3)	4.7 (1.3)	1139 (551)	469 (290)	1313 (669)	533 (339)	7.5 (1.7)	6.8 (1.8)
HO-Bupropion											
P	normal	542 (145)	26.7 (2.6)	12.3 (5.2)	8.4 (1.7)	13778 (4205)	567 (81.7)	n.d.	n.d.	n.d.	n.d.
P	reduced	358 (53.3)	18.6 (6.2)	16.9 (7.6)	9.6 (3.3)	9082 (1685)	417 (131)	n.d.	n.d.	n.d.	n.d.
M	normal	479 (180)	29.4 (13.6)	13.0 (7.2)	7.8 (2.7)	11692 (3122)	521 (187)	n.d.	n.d.	n.d.	n.d.
M	reduced	452 (180)	29.6 (10.0)	11.7 (4.6)	8.5 (1.4)	10527 (5561)	626 (212)	n.d.	n.d.	n.d.	n.d.

## Discussion

The co-administration of CYP2D6 inhibitors was used to mimic an IM or PM genotype, which is a feasible approach to study the influence of CYP2D6 on MDMA metabolism. Previous studies used paroxetine as a CYP2D6 inhibitor in seven EMs who were orally administered MDMA [39, 51]. However, the analysis was only performed with racemic MDMA and free metabolites after conjugate cleavage. As not only stereoselectivity of MDMA itself, but also of its primary metabolite DHMA is discussed in terms of (neuro-)toxicity [52, 53] the further metabolic fate of free DHMA and its resulting stereoselectivities is of interest. Stereoselectivity is not only obtained from initial formation but rather influenced by follow-up metabolic steps such as phase II metabolism. Furthermore, recent studies discussed DHMA to play a role in acute cardiovascular effects observed after MDMA consumption [54]. It was shown that the initial CYP2D6 metabolite DHMA is not present in human plasma in its free form but rather as its sulfate conjugates [23, 55], thus making meaningful interpretations of the impact of CYP2D6 difficult because at least one additional metabolic step occurs. Such an analysis after conjugate cleavage will not represent the actual abundance of all metabolites that are present in plasma, thus complicating correlations with potential neurotoxicity. Furthermore, CYP2D6 inhibition might result in higher activity of other CYP enzymes with different stereoselectivity, leading to differences in *R*- and *S*-MDMA and consequently different pharmacodynamic effects and acute toxicity [1, 2, 9, 33, 56]. Therefore, the present study sought to elucidate the effects of bupropion in mimicking a reduction of CYP2D6 activity in humans and its influence on the metabolism and pharmacokinetics of MDMA in a stereoselective manner and after direct analysis of the conjugates. Although from the pharmacological and toxicological point of view no relevant toxicity is expected from the phase II metabolites as terminal metabolites, their (stereoselective) formation should majorly influence the concentration and stereoselectivity of the active metabolite DHMA. Bupropion similarly inhibited CYP2D6 as paroxetine and fluoxetine *in vivo*, but it is not a substrate of CYP2D6 [44]. The data could then be compared for different genotypes of CYP2D6 (EM and IM).

We found that the pharmacokinetics of MDMA and MDA enantiomers in EMs were comparable to those published in other studies [36, 57].

Bupropion increased *R*- and *S*-MDMA C_max_ and AUC_24_ values in CYP2D6 EMs resulting in exposure to MDMA in the same range as previously described for racemic MDMA in one PM [58] and after CYP2D6 inhibition with paroxetine [39]. The t_max_ and t_onset_ of both enantiomers were unaltered by bupropion, whereas t_1/2_ was prolonged and plasma clearance was reduced, suggesting that the metabolic interaction was responsible for differences in pharmacokinetics rather than absorption and/or distribution, which is again consistent with other studies [39].

*R-* and *S-*MDA C_max_ and AUC_24_ values were decreased by the co-administration of bupropion. MDA is mainly formed through CYP2B6, with bupropion as a CYP2B6 substrate [59] that acts as a competitive inhibitor in this pathway. In CYP2D6 IMs, *S-*MDA levels were not significantly different from EMs with regard to either C_max_ or AUC_24_ (Fig 1A). This indicates the competitive inhibition of CYP2B6 by bupropion. Segura et al. reported an opposite effect with racemic MDA, in which C_max_ and AUC levels increased, thus suggesting accumulation through the inhibition of subsequent MDA pathways by CYP2D6 [39].

As expected, the co-administration of bupropion and MDMA decreased all metabolites that are formed CYP2D6-dependently. Although the extent of CYP2D6-inhibition by bupropion on C_max_ and AUC_24_ values was comparable for *R*- and *S*-DHMA, 3-sulfate, *R-/S-*DHMA 4-sulfate, and *R-/S-*HMMA sulfate, the reduction of *S*-HMMA glucuronide was significantly greater. UGTs are enzymes with low affinity for their substrates but high capacity, whereas SULTs have high affinity for their substrates but are easily saturated. If lower concentrations of HMMA are present as a consequence of a decrease in the CYP2D6-dependent formation of DHMA and consequently HMMA, then the higher affinity of SULT should be more relevant than the higher capacity of UGTs. Furthermore, UGT inhibition by bupropion might occur [60]. Although no significant differences in C_max_ and AUC_24_ were found between the bupropion-pretreated CYP2D6 EMs and placebo-pretreated IMs, the *S*-enantiomer C_max_ of sulfate metabolites appeared to be lower after bupropion pretreatment in EMs compared with IMs who were pretreated with placebo. This effect was not observed for HMMA glucuronide. Notably, the present study only included three IMs. The statistical power, therefore, was rather low, and true differences might have gone undetected. The t_max_ of all of the conjugates and t_onset_ for *R*- and *S*-HMMA sulfate and *R*- and *S*-HMMA glucuronide were reached significantly later with bupropion compared with placebo pretreatment, which could be congruent with lower first-pass metabolism. Additionally, t_1/2_ was substantially prolonged after bupropion pretreatment compared with placebo pretreatment, but it was not significantly different between CYP2D6 IMs and EMs. This suggests that other than CYP2D6-mediated interactions might be involved.

Other CYP enzymes might compensate for the decrease in CYP2D6 activity, and different *R-* and *S-* stereoisomer concentrations and thus pharmacological effects might occur due to different potencies of enantiomers[3–6, 52, 53]. For MDMA, significant reductions of *R/S* ratios were observed between placebo-pretreated CYP2D6 EMs and bupropion-pretreated EMs but not between placebo-pretreated CYP2D6 IMs and bupropion-pretreated EMs over 24 h (Fig 4). CYP2D6 preferentially demethylenates *S-*MDMA, and the inhibitory effect should be more dominant on that enantiomer, resulting in higher concentrations of the pharmacologically more active *S*-MDMA through a reduction of metabolism. The inhibition of CYP2D6 is likely to result in the loss of metabolic enantioselectivity because other enzymes do not provide comparable stereoselectivity as CYP2D6 [26]. However, the observed mean overall effect on MDMA *R/S* ratios was less than 10%. MDA showed the same effect, likely because of inhibition of its metabolism by CYP2D6, which is identical to MDMA. Importantly, MDMA is a CYP2D6 inhibitor [61], and only one high dose of MDMA was administered, similar to a previous study [39]. Phase II metabolites represent secondary or even tertiary metabolites that involve further enzymes. Generally, t_1/2_ values for *R*-enantiomers were higher than for *S-*stereoisomers [23]. After pretreatment with bupropion, the prolongation of t_1/2_ for *R*-conjugates was even higher than for *S-*enantiomers.

High interindividual variations in plasma concentrations of MDMA and its metabolites have been reported [23]. The questions how CYP2D6 genotypes or CYP2D6 inhibition alter concentrations of MDMA [61–63] is of interest in clinical and forensic toxicology. Different approaches were considered in the present study to address this question. For *R/S* ratios, only slight differences between CYP2D6 EMs, IMs, and bupropion-pretreated EMs were observed. In contrast, significant differences were found for metabolite ratios of CYP2D6-dependetly formed metabolites to MDMA between groups (Fig 5a, exemplified by the *R-*DHMA 3-sulfate/*R*-MDMA ratio). However, large variations depending on the time post MDMA dose (Fig 5b) were observed limiting the value of these ratios as useful estimation of CYP2D6 function without knowledge of last MDMA intake in forensic cases. For all conjugates, initial high values were observed in CYP2D6 EMs, which can be explained by rapidly occurring first-pass metabolism. This effect was nearly completely lost in CYP2D6 IMs and after co-administration of the CYP2D6 inhibitor bupropion, which is consistent with lower first-pass metabolism. Oftentimes, correlations between genotypes and phenotypes are based on AUC values or AUC ratios between metabolites and/or parent compounds. However, in these studies, multiple blood samples and full pharmacokinetic analysis are necessary. Nevertheless, AUC_24_ ratios between MDMA conjugates and MDMA correlated well with CYP2D6 genotype and bupropion-inhibited phenotype (Fig 6).

Bupropion and its main metabolite hydroxybupropion were analyzed stereoselectively in the present study to determine the effect of MDMA on bupropion`s complex and stereoselective metabolism and possible correlations with CYP2D6 inhibition. The minor dehydrometabolite was previously determined in a pharmacodynamics study [40] and thus not included in the present study. The t_1/2_ of hydroxybupropion diastereomers could not be determined because of slow elimination and the limited blood sampling time of only 24 h. Plasma concentration-time profiles were comparable to those in previous studies after a single bupropion dose [43]. No stereoisomer of bupropion or hydroxybupropion was significantly different between the bupropion-placebo and bupropion-MDMA groups (Fig 7), indicating that the CYP2D6 inhibitor MDMA had no influence on bupropion metabolism. This was expected because bupropion is primarily hydroxylated by CYP2B6 [59]. Although MDMA is also partially cleared by CYP2B6 to MDA, this only represents a minor pathway and is somewhat in contrast to a previous racemic analysis of the same samples, showing increases in both bupropion and hydroxybupropion through MDMA. However, the observed effect could not be fully explained [40] and was not reproducible in the chiral reanalysis.

No correlation was found between *R-* or *S-*bupropion concentrations and the CYP2D6-dependently formed MDMA metabolites. Previous studies suggested that bupropion’s metabolites (mainly dehydrobupropion) rather than bupropion itself act as CYP2D6 inhibitors [45]. However, comparisons of *R*,*R*-hydroxybupropion, *S*,*S*-hydroxybupropion, and dehydrobupropion did not show such a relationship.

## Conclusion

The CYP2D6 inhibitor bupropion altered the chiral pharmacokinetics of MDMA. Although interactions other than the metabolic inhibition of CYP2D6 seem to occur, generally good agreement was found between the effects of genetically impaired CYP2D6 function (EMs *vs*. IMs) and the effects of pharmacological CYP2D6 inhibition (placebo *vs*. bupropion-pretreated EMs) on the metabolism of MDMA. CYP2D6 function modulated exposure to MDMA *R*- and *S-*enantiomers, but the clinical implications of this pharmacokinetic interaction remain to be determined. The present pharmacokinetic data might aid in further interpretations of toxicity and neurotoxicity based on CYP2D6-dependent MDMA metabolism and resulting stereoselective concentrations.

## Supporting Information

S1 Fig Chiral LC-MS/MS analysis of bupropion and hydroxybupropion. MRM chromatogram of the chiral analysis of bupropion and hydroxybupropion on a Chiral AGP column. Depicted are the quantifier MRM chromatograms of R/S-bupropion and R,R-/S,S-hydroxybupropion of a QC med sample.
(TIF)

S2 Fig Consort flow chart. Flow diagram of the progress through the phases of the randomized clinical trial including enrolment, intervention allocation, follow-up, and data analysis.
(PPT)

S1 Table Validation data. Validation data for chiral bupropion analysis, RE: recovery; CV: coefficient of variation; ME: matrix effect; IS: internal standard; RSDR: intraday precision; RSDT: interday precision; QC: quality control.
(DOCX)

S1 Text Chiral analysis of bupropion and hydroxybupropion. (DOCX)

S2 Text Study protocol. (PDF)
