# Supplementary figures and images for: Impact of Cytochrome P450 2D6 Function on the Chiral Blood Plasma Pharmacokinetics of 3,4-Methylenedioxymethamphetamine (MDMA) and Its Phase I and II Metabolites in Humans
Source: PLoS One. 2016 Mar 11;11(3):e0150955. doi: 10.1371/journal.pone.0150955 (PMC4788153; doi:10.1371/journal.pone.0150955)

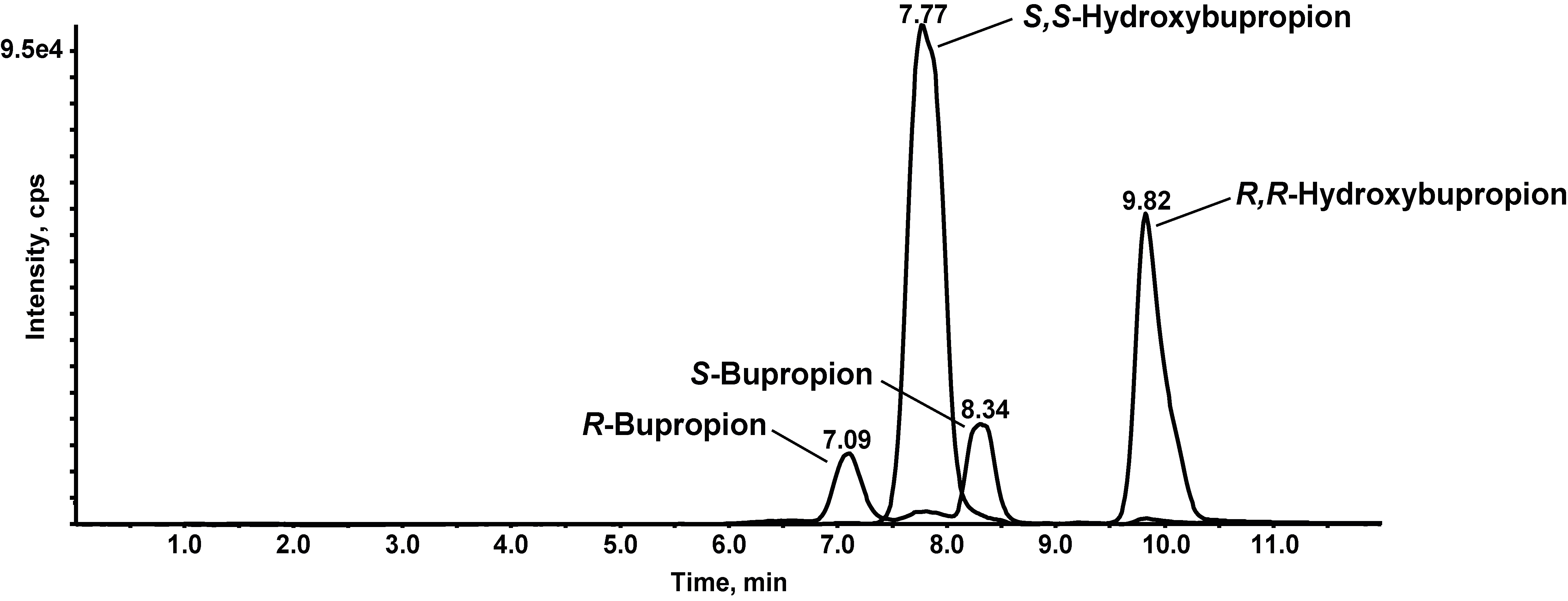

Supplement: S1 Fig — MRM chromatogram of the chiral analysis of bupropion and hydroxybupropion on a Chiral AGP column. Depicted are the quantifier MRM chromatograms of R/S-bupropion and R,R-/S,S-hydroxybupropion of a QC med sample. (TIF) [file pone.0150955.s001.tif]
