# Supplementary material for: Impact of Cytochrome P450 2D6 Function on the Chiral Blood Plasma Pharmacokinetics of 3,4-Methylenedioxymethamphetamine (MDMA) and Its Phase I and II Metabolites in Humans
Source: PLoS One. 2016 Mar 11;11(3):e0150955. doi: 10.1371/journal.pone.0150955 (PMC4788153; doi:10.1371/journal.pone.0150955)
Supplement: S2 Fig — Flow diagram of the progress through the phases of the randomized clinical trial including enrolment, intervention allocation, follow-up, and data analysis. (PPT) [file pone.0150955.s002.ppt]

## Slide 1
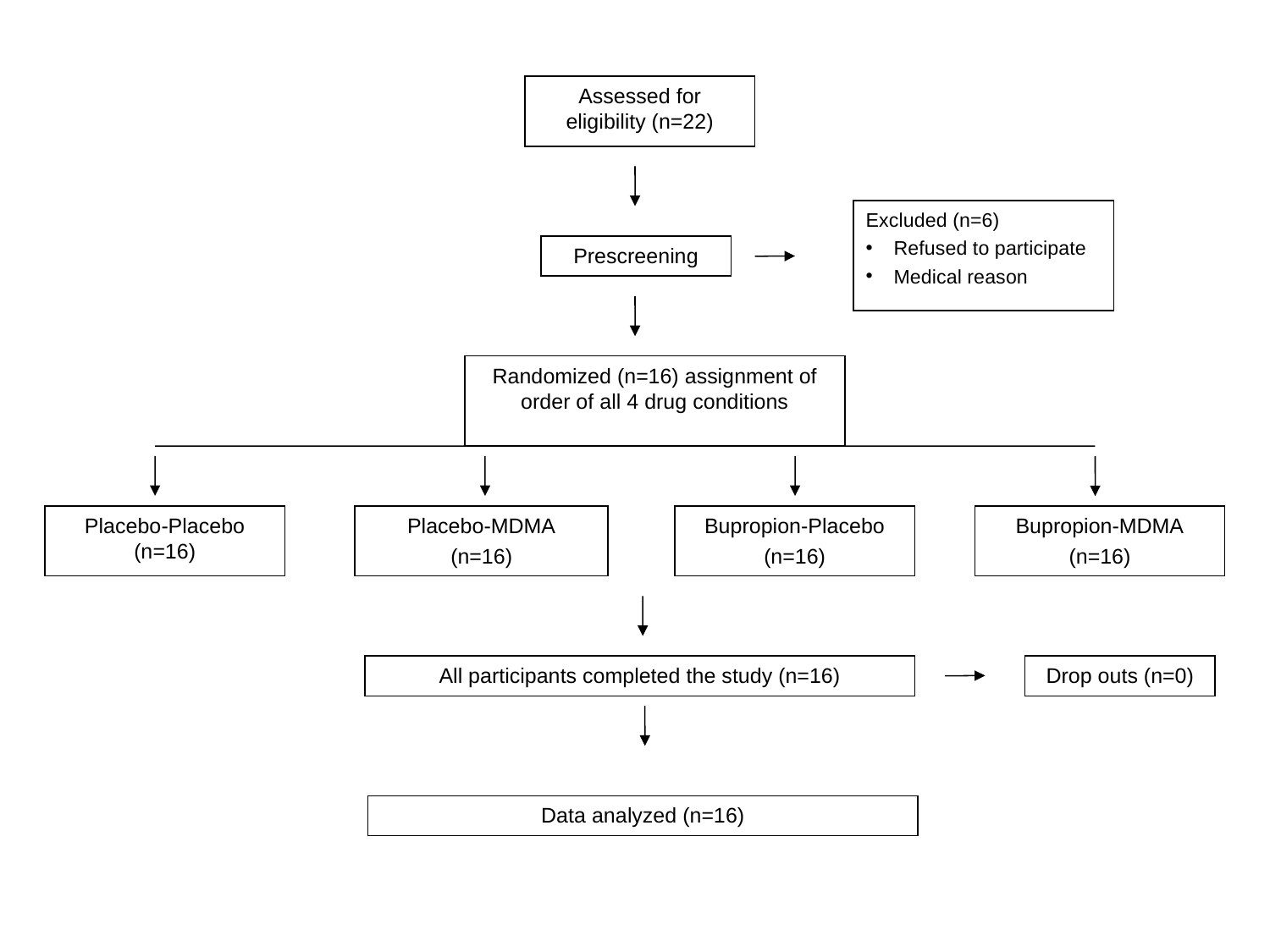

# Assessed for eligibility (n=22)
Excluded (n=6)
Refused to participate
Medical reason
Prescreening
Randomized (n=16) assignment of order of all 4 drug conditions
Placebo-Placebo (n=16)
Placebo-MDMA
(n=16)
Bupropion-Placebo
(n=16)
Bupropion-MDMA
(n=16)
All participants completed the study (n=16)
Drop outs (n=0)
Data analyzed (n=16)
