# Supplementary material for: Impact of Cytochrome P450 2D6 Function on the Chiral Blood Plasma Pharmacokinetics of 3,4-Methylenedioxymethamphetamine (MDMA) and Its Phase I and II Metabolites in Humans
Source: PLoS One. 2016 Mar 11;11(3):e0150955. doi: 10.1371/journal.pone.0150955 (PMC4788153; doi:10.1371/journal.pone.0150955)
Supplement: S1 Table — Validation data for chiral bupropion analysis, RE: recovery; CV: coefficient of variation; ME: matrix effect; IS: internal standard; RSDR: intraday precision; RSDT: interday precision; QC: quality control. (DOCX) [file pone.0150955.s003.docx]

**Table S1:** **Validation data for chiral bupropion analysis**

RE: recovery; CV: coefficient of variation; ME: matrix effect; IS internal standard; RSD_R_: intraday precision; RSD_T_: interday precision; QC quality control

| Analyte | QC level | QC conc.  ng/ml | RE, % (CV, %) | RE, % (CV, %)  (IS corrected) | ME, % (CV, %) | ME, % (CV, %)  (IS corrected) | Bias, % | RSD_R,_ % | RSD_T,_ % |
| --- | --- | --- | --- | --- | --- | --- | --- | --- | --- |
| *R*-Bupropion | QC low | 6 | 67.2 (5.5) | 105.1 (3.9) | 248.4 (1.3) | 99.7 (5.7) | 5.9 | 6.7 | 9.5 |
|  | QC med | 50 |  |  |  |  | 0.4 | 4.8 | 4.5 |
|  | QC high | 175 | 68.6 (14.4) | 98.7 (2.8) | 219.9 (2.7) | 99.3 (2.5) | 6.7 | 2.4 | 5.6 |
| *S*-Bupropion | QC low | 6 | 71.0 (2.5) | 109.9 (4.9) | 242.7 (7.6) | 102.8 (8.6) | 9.2 | 3.7 | 9.6 |
|  | QC med | 50 |  |  |  |  | 0.5 | 6.0 | 5.6 |
|  | QC high | 175 | 69.0 (10.8) | 99.3 (3.8) | 192.4 (6.9) | 101.7 (2.2) | 3.4 | 2.8 | 5.5 |
| *R,R*-HO-Bupropion | QC low | 3.25 | 73.2 (13.3) | 116.3 (7.1) | 15.9 (12.5) | 37.8 (17.1) | 4.3 | 7.0 | 13.9 |
|  | QC med | 200 |  |  |  |  | 8.3 | 7.8 | 7.5 |
|  | QC high | 750 | 61.2 (38.7) | 79.9 (16.3) | 69.8 (18.6) | 112.2 (14.5) | -7.5 | 10.1 | 13.9 |
| *S,S*-HO-Bupropion | QC low | 3.25 | 69.7 (2.4) | 82.0 (1.9) | 16.9 (1.3) | 86.3 (11.7) | 8.4 | 1.8 | 11.4 |
|  | QC med | 200 |  |  |  |  | 14.2 | 3.6 | 5.0 |
|  | QC high | 750 | 80.8 (5.5) | 94.3 (2.1) | 95.1 (2.4) | 100.1 (2.7) | -2.6 | 1.7 | 6.6 |
