# Supplementary material for: Impact of Cytochrome P450 2D6 Function on the Chiral Blood Plasma Pharmacokinetics of 3,4-Methylenedioxymethamphetamine (MDMA) and Its Phase I and II Metabolites in Humans
Source: PLoS One. 2016 Mar 11;11(3):e0150955. doi: 10.1371/journal.pone.0150955 (PMC4788153; doi:10.1371/journal.pone.0150955)
Supplement: S1 Text — (DOCX) [file pone.0150955.s004.docx]

**S1 Text. Chiral analysis of bupropion and hydroxybupropion**

*Sample preparation*

Blood plasma samples were analyzed stereoselectively for *R-* and *S-*bupropion and its major metabolites *R,R-* and *S,S-*hydroxybupropion according to ref. [[38](#_ENREF_37)] with slight modifications. 200 µl plasma were mixed with 20 µl of the internal standard (IS) mixture (bupropion-d9 1 µg/ml, hydroxybupropion-d6 5µg/ml), 600 µl of acetonitrile were added; the mixture was shaken and centrifuged at 10,000g for 5 min). An aliquot of 550 µl was transferred into an autosampler vial, 50 µl of formic acid were added and the mixture was evaporated to dryness under a gentle stream of nitrogen at 40 °C. The residue was dissolved in 100 µl of mobile phase 20 mM ammonium acetate buffer, pH 6.6 (A) and methanol (B) (1:1, v/v). 10 µl of this solution were injected into the LC-MS/MS system.

*LC-MS/MS analysis*

The analysis was performed using a Thermo Fischer Ultimate 3000 UHPLC system (Thermo Fisher, San Jose, California, USA) coupled to an ABSciex 5500 QTtrap linear ion trap quadrupole mass spectrometer (ABSciex, Darmstadt/Germany) with positive electrospray ionization (ESI) in multiple reaction monitoring mode (MRM).

The LC settings were as follows: Chiral AGP column (100 x 4.0 mm, 5 µm), gradient elution with 20 mM ammonium formate buffer in water (pH 6.6, A) and methanol (B) . The flow rate was 0.8 ml/min with the following gradient: start conditions 10% B for 0.5 min, 0.5-6 min to 30% B, 6-7 min to 50% B, 7-9 min to 80% B hold at 80% B for 1 min, at 10 min reequilibrating to 10% B for 2 min. Injection volume was 10 µl.

The Turbo V ion source equipped with a stainless steel electrode (100 µm internal diameter) was operated in positive ESI mode with the following MS conditions: gas 1, nitrogen (50 psi); gas 2, nitrogen (60 psi); ion spray voltage, 5500; ion-source temperature, 550 °C; curtain gas, nitrogen (30 psi), collision gas, high. The MS was operated in the MRM mode with a scan time of 50 ms using 3 transitions for each analyte except for the ISs where 1 MRM transition was used. The following MRM transitions (declustering potential DP, entrance potential EP, collision energy CE, cell exit potential CXP) were used with quantifiers given in bold: *R-/S*-bupropion **240 🡪 184** (56, 10, 19, 10), 240 🡪 166 (121, 10, 25, 10), 240 🡪 131 (121, 10, 25, 12) and *R,R-/S-,S-* hydroxybupropion **256 🡪 238** (51, 10, 17, 10), 256 🡪 139 (1, 10, 35, 10). Respective deuterated analogues were used as internal standards with the following MRM transition bupropion d9 **249 🡪 185** (61, 10, 27, 10) and hydroxybupropion d6 **262 🡪 244** (56, 10, 19, 10). The MS was controlled by analyst 1.6.2 software.
