# Supplementary material for: Impact of Cytochrome P450 2D6 Function on the Chiral Blood Plasma Pharmacokinetics of 3,4-Methylenedioxymethamphetamine (MDMA) and Its Phase I and II Metabolites in Humans
Source: PLoS One. 2016 Mar 11;11(3):e0150955. doi: 10.1371/journal.pone.0150955 (PMC4788153; doi:10.1371/journal.pone.0150955)
Supplement: S2 Text — (PDF) [file pone.0150955.s005.pdf]

Influence of Bupropion on the Effects of MDMA

Protocol: Version 2, 30.7.2012

Principal Investigator/verantwortlicher Prüfer

PD Dr. Matthias E. Liechti, MD, MAS

30.7.2012 …………………….
date, signature

Division of Clinical Pharmacology and Toxicology
University Hospital Basel
Hebelstrasse 2
CH-4031 Basel
Switzerland

Fax	++41 61 265 45 60
Phone	++41 61 328 68 68
E-mail	mliechti@uhbs.ch

1 SYNOPSIS

Sponsor	Clinical Pharmacology & Toxicology, University Hospital Basel
Study title	Influence of bupropion on the effects of MDMA
Principal investigator	PD Dr. med. Matthias Liechti
Study site	Divisions of Clinical Pharmacology & Toxicology, University Hospital Basel
Objectives	**Primary study endpoints**<br>1. Reduction of the positive mood response to MDMA by bupropion<br>**Secondary study endpoints**<br>1. Effect of bupropion on the cardiovascular response to MDMA<br>2. Effects of bupropion on the endocrine response to MDMA<br>3. Effect of bupropion on the pharmacokinetics of MDMA<br>4. Effect of personality traits and genetic polymorphisms on the response to MDMA<br>5. Tolerability of MDMA and bupropion
Design	Randomized, double-blind, placebo-controlled, 4-period cross-over study
Subjects	16 healthy volunteers (8 men, 8 women), age: 18-45 years
Drugs	Bupropion (Wellbutrin XR®, 150 mg p.o.) and MDMA (3,4-methylenedioxy-methamphetamine, 125 mg p.o.)
Study dates	Start: 1.10.2012, End: 30.9.2013

2 INDEX

1 SYNOPSIS 2
2 INDEX 3
3 Abbreviations and Definitions 5
4 SUMMARY 6
5 INTRODUCTION 7
6 SIGNIFICANCE 8
7 BACKGROUND 9
7.1 Role of DA in the effects of MDMA 9
7.2 Expected interactive effects of bupropion and MDMA 9
7.2.1 Pharmacodynamic interactions 9
7.2.2 Pharmacokinetic interactions 9
8 STUDY OBJECTIVES 9
8.1 Primary study endpoints 9
8.2 Secondary study endpoints 10
9 HYPOTHESES 10
9.1 Primary Hypotheses 10
9.2 Secondary Hypotheses 10
10 STUDY DESIGN AND METHODS 10
10.1 Study design 10
10.2 Study duration 10
10.3 Study site 10
10.4 Study population 10
10.4.1 Recruitment 10
10.4.2 Inclusion criteria 10
10.4.3 Exclusion criteria 11
STUDY PROCEDURES 11
10.5 Schedule of Events 11
10.6 Screening procedure 11
10.6.1 Informed consent 11
10.6.2 Physical health 11
10.6.3 Mental health 11
10.6.4 History of Drug use 12
10.6.5 Screening laboratory tests 12
10.6.6 CYP2D6 phenotyping 12
10.6.7 Genotyping 12
10.6.8 Personality 12
10.6.9 Menstrual cycle phase in women 12
10.7 Schedule of experimental session 12
10.8 Assessments and Measures 13
10.8.1 Psychometric assessments 13
10.8.1.1 Visual Analog Scales (VAS) 13
10.8.1.2 Adjective mood rating scale (AMRS) 13
10.8.1.3 Addiction Research Center Inventory (ARCI) 13
10.8.1.4 Altered states of consciousness (5D-ASC) 13
10.8.2 Physiological assessments 14
10.8.2.1 Vital signs 14
10.8.2.2 List of complaints (LC) and Adverse effects (AE) 14
10.8.2.3 Emotion recognition 14
10.8.2.4 Empathy and social value orientation 15
10.8.2.5 End of Session Questionnarie 15
10.8.3 Neuroendocrine function 15
10.8.4 Blood sample collection (pharmacokinetics) 15
10.8.4.1 Amount of blood samples 16
10.8.5 End of Study (EOS) visit 16
10.8.5.1 EOS Examination 16
10.8.5.2 EOS Questionnaire 16
10.9 Study Drugs 16
10.9.1 Bupropion 16

10.9.2 MDMA ... 17
10.9.2.1 Chemistry, manufacturing, control ... 17
10.9.2.2 Metabolism and Pharmacokinetics of MDMA ... 17
10.9.2.3 Dose selection (MDMA) ... 17
10.9.3 Drug accountability, storage and return of study drugs ... 17
10.9.4 Concomitant medications ... 17
10.9.5 Randomization and blinding ... 17
10.9.6 Compliance ... 18
11 Analytics ... 18
11.1 MDMA and metabolites ... 18
11.2 CYP2D6 Phenotyping ... 18
11.3 Cortisol, prolactine, progesterone, estradiol, testosteron ... 18
11.4 Catecholamines and metanephrines ... 18
11.5 Copeptin ... 18
11.6 Oxytocin ... 18
12 Data analysis ... 18
12.1 Sample size estimation ... 18
12.2 Analysis of outcomes ... 18
13 Protection of subjects ... 19
13.1 Potential risks ... 19
13.1.1 Specific toxicity of MDMA and monitoring ... 19
13.2 Risk-Benefit Assessment ... 20
13.3 Monitoring of toxicity ... 21
13.3.1 Safety definitions ... 21
13.3.2 Documentation ... 22
13.3.3 Adverse events (AE) documentation ... 22
13.3.4 SAE and SUSAR reporting ... 22
13.3.5 Medical follow-up of adverse events ... 22
13.4 Ethical standards ... 22
13.4.1 Institutional Review Board ... 22
13.4.2 Protocol amendments ... 22
13.4.3 Early study termination ... 22
13.4.4 Insurance ... 23
13.4.5 Compensation ... 23
13.4.6 Premature withdrawal of subjects ... 23
13.4.7 Replacement policy ... 23
13.5 Legal authorizations ... 23
13.6 Trial registration ... 23
13.7 Study documentation and record keeping ... 23
13.8 Quality control and quality assurance ... 23
13.8.1 Training of personnel and SOPs ... 23
13.8.2 Monitoring ... 24
13.8.3 Direct access to source data ... 24
13.8.4 Inspection ... 24
13.8.5 Confidentiality ... 24
14 Time plan ... 24
15 Publications ... 24
16 Budget and Funding ... 24
17 Certifications of principal investigator ... 24
18 Conflict of interest ... 24
19 Research Environment ... 24
19.1 Study site ... 24
20 Responsibilities of the sponsor-investigator ... 24
21 References ... 25

3 Abbreviations and Definitions

ASAT	aspartate transferase
ALAT	alanine aminotransferase
AE	adverse event
ANOVA	analysis of variance
AMRS	adjective mood rating scale
AR	adverse reaction
AUC	area under the concentration-time-curve
BAG	Bundesamt für Gesundheits-wesen, Swiss Federal Office of Public Health
BP	blood pressure
CHF	Swiss francs
CRF	case report form
CRP	C-reactive proteine
CTU	Clinical Study Coordination Center / Clinical Trial Unit of the University Hospital Basel
CYP	cytochrome P450 enzyme
C_{max}	maximum concentration
Cl	chloride
Ca	calcium
5D-ASC	5 dimensions of altered states of consciousness
DA	dopamine
DSM-IV	Diagnostic and Statistical Manual Version 4
ECG	electrocardiogram
EWL	Eigenschaftswörterliste, AMRS
Ecstasy	may contain MDMA or other substances at various amounts
EKBB	Ethische Kommission beider Basel, IRB
ECG	electrocardigram
EOS	end of study
FPI	Freiburger Personality Inventory
GCP	good clinical practice
GC-MS	gas chromatography-mass spectrometry
GMP	good manufacturing practice
γ-GT	gamma-glutamyl transferase
HPLC	high-pressure liquid chromato-graphy
5-HT	serotonin
HR	heart rate
HMA	4-hydroxy-3-methoxy-amphetamine
HMG	Heilmittelgesetz
HMMA	4-hydroxy-3-mehoxymeth-amphetamine
5-HIAA	5-hydroxy indole acetic acid
ICH	International Conference on Harmonization
IRB EKBB	institutional ethics board =
K	potassium
LSD	lysergic acid diethylamine
LC	list of complaints
LDH	laccate dehydrogenase
LC-MS/MS	liquid chromatography-tandem mass spectroscopy
MDMA	3,4-Methylenedioxymeth-amphetamine
MDA	3,4-methylenedioxy-amphetamine
MD	medical doctor
Na	sodium
NE	norepinephrine, noradrenaline
NEO-FFI	NEO-Fünf Faktoren Inventar
P	phosphorus
PD	pharmacodynamics
PK	pharmacokinetic
PI	principal investigator
PRU	phase 1 research unit
P_{sys}	systolic blood pressure
PhD	Philosophical doctor (Dr. Phil)
THC	tetrahydrocannabinol
SAE	serious adverse event
SSRI	selective serotonin uptake inhibitor
SD	standard deviation
SUSAR	Suspected Unexpected Serious Adverse Reaction
$T_{1/2}$	Plasma eliminiation half-life
T_{max}	time to reach C_{max}
UAR	unexpected adverse reaction
VAS	visual analogue scale
Vklin	Verordnung über klinische Versuche mit Heilmitteln

4 SUMMARY

3,4-Methylenedioxymethamphetamine (MDMA, “ecstasy”) is used as recreational drug because of its positive mood effects. MDMA releases serotonin (5-HT), dopamine (DA), and norepinephrine (NE) via the corresponding transporter. DA mediates the reinforcing addiction-related effects of drugs of abuse but it is unclear whether DA contributes to the acute effects of MDMA in humans. Therefore, we plan to investigate the role of DA transporter-mediated DA release in the acute response to MDMA in humans using the DA transporter inhibitor bupropion (Wellbutrin) as a pharmacological tool. We will investigate the effects of a pretreatment with bupropion (300 mg once-daily) or placebo on the acute effects of MDMA (125 mg) or placebo in 16 healthy subjects using a double-blind placebo-controlled randomized four-period cross-over design. The primary outcome is the reduction in MDMA-induced positive mood by bupropion. Secondary outcome measures include vital signs, hormones, pharmacokinetics, and tolerability. The study will enhance our understanding of the dopaminergic regulation of mood and may help in the development of treatments for stimulant addiction.

Key words: MDMA, 3,4-Methylenedioxymethamphetamine, ecstasy, dopamine, bupropion, pharmacodynamics, pharmacokinetics

5 INTRODUCTION

3,4-Methylenedioxymethamphetamine (MDMA, “ecstasy”) is widely used as a recreational drug. MDMA interacts with monoamine transporters to release presynaptic serotonin (5-HT), dopamine (DA), and norepinephrine (NE). Serotonin and NE have both been shown to mediate aspects of the acute psychotropic and physiological effects of MDMA in humans [1-4]. However, the role of DA in the acute mechanism of action of MDMA is not clear. Here, we suggest evaluating the functional role of DA in the pharmacology of MDMA in humans using the DA transporter (DAT) inhibitor bupropion as a pharmacological tool compound. We plan to test the effects of a pretreatment bupropion on the pharmacodynamics and pharmacokinetics of MDMA in healthy human subjects. We hypothesize that bupropion will significantly reduce the subjective response to MDMA. Such a result would indicate that DAT-mediated DA release contributes to the psychotropic effects of MDMA.

The study will enhance our knowledge of the regulation of mood. A better understanding of the role of DA in the acute psychological effects of psychoactive drugs will also help in the development of treatments for stimulant addiction. The study is part of a series of clinical studies to evaluate the role of monoamines in the mechanisms of action of amphetamine-type stimulants in humans and is financially supported by the Swiss National Foundation (SNF grant: 323230_126231/1 to PD Dr. Matthias Liechti). We have conducted several comparable studies as part of this SNF project (EKBB373/08 [3], EKBB253/09, EKBB353/09 [6], EKBB218/10 [7], EKBB65/11 , EKBB228/11).

Bupropion is an antidepressant with an amphetamine-like structure which inhibits the DAT and to a lower extent also the NET [8]. In terms of its pharmacological action bupropion has similarities with cocaine and amphetamines which are also monoamine uptake inhibitors. However, bupropion, does not produce the pronounced stimulant and euphoric effects of the recreationally used drugs [9]. Bupropion is widely used to assist smoking cessation and as an antidepressant. Bupropion is also a candidate medication for the treatment of methamphetamine dependence [10]. Bupropion pretreatment (150 mg twice-daily for 6 days) reduced the subjective drug high following methamphetamine administration in a laboratory study [10]. In addition, two clinical trials showed that bupropion reduced methamphetamine use, mostly in subjects with low baseline methamphetamine use [11-14]. Bupropion inhibits DA release produced by amphetamines. Specifically, bupropion increased the EC_{50} value of DAT-mediated amphetamine-induced DA release from rat synaptosomes from 0.07 to 2.5 μM indicating competitive inhibition [15]. Bupropion also inhibited methamphetamine- or MDMA-induced DA efflux from human DAT-transfected DA preloaded HEK293 cells (unpublished data, Wandeler, Simmler, and Liechti). The *in vitro* data and the results from the clinical trials indicate that bupropion could be an ideal probe drug to inhibit the effects of MDMA in humans to the extent that they depend on DAT-mediated DA release. We have previously used the DAT and NE transporter (NET) inhibitor methylphenidate (MPH) to similarly inhibit effects of MDMA in healthy subjects. We found that both MPH and MDMA produced cardio- and psychostimulant effects when administered alone with no further increase when both drugs were administered together. Thus, MPH and MDMA had interactive effects suggesting a partly common mode of action. However, because MPH had psychotropic effect on its own that were to some extent similar to those of MDMA we could not demonstrate an inhibition of the response to MDMA. The use of bupropion in the present study has several advantages. First, bupropion is expected to have no relevant psychotropic effects on its own, unlike MPH. Second, bupropion is a 7-fold more potent DAT than NET inhibitor and therefore more selective for DAT compared to MPH which blocks DAT and NET with equal potency. Thus, the pharmacological “tool” bupropion will allow us to more selectively study the role of DA in the mechanism of action of MDMA in humans. The potency (K_m) of bupropion to inhibit the human DAT is 1.8 μM compared with 0.15 μM for MPH (Wandeler, unpublished data). This means that bupropion is approximately 10-fold less potent than MPH as DAT inhibitor, however, bupropion is used in clinical doses resulting in plasma concentrations that are 10-fold higher than those obtained with MPH [8]. Bupropion reaches a high brain-to-plasma ratio and brain concentrations above its IC_{50} value for DAT inhibition when administered in clinical doses [8]. Administration of 150 mg bupropion twice-daily, as proposed for this study, produced DAT occupancy in the human striatum of 26% as measured 3h after the last administration of bupropion by ^{11}C-βCIT-FE PET [16]. Together the data confirm that bupropion is a DAT inhibitor in humans at clinically relevant doses [8] and that bupropion should prevent the MDMA response to the extent that effects of MDMA in humans depend on an interaction with the DAT. Our research approach also has limitations. First, bupropion also inhibits NET although to a lesser extent than DAT. Importantly, however, bupropion does not inhibit SERT and it is clearly the best currently available medication to use for our purpose. Second, it is not clear whether the DAT inhibition produced by bupropion will be sufficient to block effects of MDMA on DA release. While

bupropion inhibited effects of methamphetamine as noted above, repeated administration of bupropion did not alter subject-rated effects of cocaine in one study [17]. In another study, bupropion even enhanced the positive subjective effects of cocaine [18]. However, in the latter study bupropion was administered in a low subclinical single 200 mg dose and effective DAT occupancy was possibly not reached. In contrast, bupropion reduced cocaine use in cocaine dependent patients in a clinical trial [19] although other trials found that bupropion was ineffective [20, 21]. Taken together, bupropion may be effective in reducing effects of amphetamines which are potent DA and NE releasers but not of those of cocaine which is an uptake inhibitor of DA but also of NE and 5-HT. Several clinical trials are currently being prepared or conducted to investigate the role of DAT inhibitors including bupropion (clinical trials.gov: NCT00572234, NCT00994448, NCT00069251, and NCT00687713) and MPH (clinicaltrials.gov: NCT01044238) in the treatment of methamphetamine dependence. Our experimental laboratory study will expand this research to the mechanism of action of MDMA using a highly controlled setting and a very comprehensive set of pharmacodynamic and pharmacokinetic outcome measures.

6 SIGNIFICANCE

Use of stimulant drugs including ecstasy is highly prevalent in our society. In Switzerland, 2.2% of 15-39 year olds reported having used ecstasy at least once [22]. Our study will provide data on the effects of pure and defined doses of MDMA, the active substance usually found in ecstasy pills, in normal subjects. These data are important to interpret results from studies in Ecstasy users.

Besides from being abused as a recreational drug, MDMA is being evaluated as a treatment for post-traumatic stress disorder in several countries including Switzerland [23, 24]. Our studies will allow to better characterize the pharmacodynamics and pharmacokinetics of MDMA providing basic clinical pharmacological data for the ongoing clinical phase II trials which use similar doses of MDMA.

MDMA is also a pharmacological tool to study mood disorders due to its potential to increase empathy and rapidly elevate mood [25-27]. Research into the mechanisms of action of MDMA will provide insight into the physiology of mood and pathophysiological processes involved in mood disorders. Ecstasy use can also result in potentially fatal medical complications [28, 29].

Serious toxicity due to ecstasy use is relatively rare but may be seen in up to ten percent of patients presenting to Emergency Departments with medical problems due to ecstasy use [28]. Extensive ecstasy use has also been associated with neurotoxic effects to serotonergic brain neurons [30]. Because MDMA is widely used, a better understanding of its pharmacology and toxicology is warranted.

The proposed experimental study will generate objective, high-quality scientific information on the effects of MDMA that could not be obtained with observational studies. In particular, the present study will provide a better understanding of the role of DA in the acute psychological effects of psychoactive drugs which will help in the development of treatments for stimulant addiction.

This study will also contribute data to a large and worldwide unique cohort sample of healthy subjects with controlled MDMA use. This large sample will allow us to study research questions that can only be addressed in a larger population (n>100). Specifically, the effects of potential personality characteristics, of endocrine covariates, and of genetic polymorphisms (pharmacogenetics) on the response to MDMA will be addressed in all subjects across all studies (n=142). Similarly, the emotion recognition test data from this study will be pooled with data from our previous studies and analyzed together.

The importance of clinical research into treatment options for psychostimulant addiction is recognized by researchers and regulatory authorities as demonstrated by the increase in number of approved controlled studies in humans. This study will contribute to the efforts of finding dopaminergic medications for the treatment of stimulant dependence.

MDMA is the prototype of the “entactogen” class of recreational drugs that produce mostly positive emotional and prosocial effects [31, 32]. New designer amphetamine-type psychostimulants including the so-called “research chemicals” or “legal highs” are continuously emerging on the illicit drug market. These drugs are structurally related to MDMA and often synthesized to produce effects similar to MDMA [33, 34]. MDMA is a reference drug for these designer drugs and the study of the clinical pharmacology and toxicology of MDMA is important for our understanding of the pharmacology of the novel drugs [35].

7 BACKGROUND

7.1 Role of DA in the effects of MDMA

In animals, MDMA releases presynaptic 5-HT, NE, and DA [36-38]. The MDMA-induced release of these monoamines is due to reverse-transport of the monoamines through the corresponding transporter and can be blocked by 5-HT transporter (SERT), NET, or DAT inhibitors. For example, MPH or bupropion inhibit the MDMA-induced DA release through DAT. The role of DA in the reinforcing effects of psychostimulants is well established. For example, pretreatment with bupropion decreased methamphetamine self-administration in rhesus monkeys [39]. However, dopamine D_1, D_2, and D_3 receptor gene deletions in mice had minimal effects on MDMA-induced acute changes in locomotor behavior [40] and DAT/NET inhibition did not affect acute responses to MDMA in rhesus monkeys [41]. In humans, we have previously shown that the DA D_2 antagonist haloperidol reduced positive mood elicited by MDMA, but haloperidol depressed mood also when given alone compared to placebo [42]. Similarly, DA D_2 receptor blockade did not affect subjective responses to d-amphetamine in most studies [43, 44]. Accordingly, DA may primarily mediate the reinforcing properties of psychostimulants, but may not be the primary mediator of their acute effects [37]. In contrast to the above findings, clinical evidence indicates a role for the DAT in the reinforcing effects of psychostimulants. For example, MPH reduced intravenous amphetamine use in drug-dependent patients [45]. Bupropion also reduced the subjective methamphetamine drug high [10] and drug use in methamphetamine-dependent subjects [12, 14]. Thus, the role of DA in the mediation of the acute subjective effects of amphetamine-type stimulants in humans is not clear. In particular, the effect of selective DAT inhibition on the acute response to MDMA has not been studied in humans.

7.2 Expected interactive effects of bupropion and MDMA

7.2.1 Pharmacodynamic interactions

The interactive effect of bupropions with amphetamines including MDMA has been studied *in vitro*. Bupropion inhibited DAT-mediated amphetamine-induced DA release from rat synaptosomes [15] or methamphetamine- or MDMA-induced DA efflux from human DAT-transfected DA preloaded HEK293 cells (unpublished data, Wandeler, Simmler, and Liechti). We therefore expect that bupropion will also inhibit MDMA-induced DA release in vivo. The interactive effects of bupropion and MDMA have so far not been studied in humans. However, bupropion pretreatment, using a dosing regimen as suggested for the present study, has been shown to reduce the acute effects of methamphetamine, a structural analog of MDMA, in humans [10]. In addition, we found that MPH, another DAT inhibitor, did not enhance the cardio- or psychostimulant effects of MDMA in healthy subjects although MPH had stimulant effects on its own (unpublished data, Liechti et al.). Based on the preclinical and clinical data, we therefore expect that the pretreatment with bupropion will reduce the acute pharmacodynamic effects of a single administration of MDMA in healthy subjects in the proposed study. Bupropion will be used in doses identical to those used for smoking cessation in healthy subjects [46]. Therefore, bupropion alone will not produce relevant adverse effects or psychotropic effects in our study.

7.2.2 Pharmacokinetic interactions

Bupropion is an inhibitor of the CYP 2D6 enzyme which metabolizes MDMA. Because MDMA inhibits its own metabolism even when given alone by mechanism-based CYP 2D6 inhibition the additional effect of bupropion is expected to result in only a minimal increase in the exposure of MDMA (<20%) as previously documented for the strong CYP2D6 inhibitor paroxetine [47]. The long interval between study days of >10 day allows for CYP2D6 activity levels to return to baseline before the next study period.

8 STUDY OBJECTIVES

The study aim is to test the effect of pretreatment with bupropion on the acute effects of MDMA using a phase I pharmacokinetic-pharmacodynamic interaction study design in healthy subjects.

8.1 Primary study endpoints

1. Reduction of the positive mood response to MDMA by bupropion

8.2 Secondary study endpoints

1. Effect of bupropion on the MDMA-induced cardiovascular and endocrine effects
2. Effect of bupropion on the pharmacokinetics of MDMA
3. Effect of personality traits and genetic polymorphisms on the response to MDMA
4. Tolerability of MDMA and bupropion

9 HYPOTHESES

9.1 Primary Hypotheses

1. Bupropion pretreatment will reduce increases in positive mood scores produced by MDMA by 20% compared with MDMA alone

9.2 Secondary Hypotheses

1. Bupropion will reduce MDMA-induced elevations in systolic blood pressure by 10 mm Hg.
2. Bupropion will attenuate MDMA-induced increases in plasma NE, cortisol, and prolactin
3. Bupropion will increase the plasma exposure to MDMA (C_{max} and AUC)
4. Bupropion and MDMA will not produce serious adverse events or severe adverse effects

10 STUDY DESIGN AND METHODS

10.1 Study design

We will use a double-blind placebo-controlled cross-over design with four treatment conditions. Thus, subjects will serve as their own controls omitting within-subject variability and markedly increasing study power. The four treatment conditions are placebo-placebo, bupropion-placebo, placebo-MDMA, and bupropion-MDMA. The treatment order will be counter-balanced and pseudo-random with washout periods of at least 10 days between the test days.

10.2 Study duration

1.10.2012 to 30.9.2013

10.3 Study site

Phase 1 Unit of the University Hospital of Basel, Switzerland.

10.4 Study population

10.4.1 Recruitment

Subjects will be recruited by word of mouth and by advertisements placed on advertising boards of university institutes of the University of Basel and on the homepage of the University. Sixteen male or female subjects will be enrolled. Drop-outs will be replaced.

10.4.2 Inclusion criteria

1. Age between 18 and 45 years
2. Understanding of the German language
3. Understanding the procedures and the risks associated with the study
4. Participants must be willing to adhere to the protocol and sign the consent form
5. Participants must be willing to refrain from taking illicit psychoactive substances during the study.
6. Participants must be willing to drink only alcohol-free liquids and no xanthine-containing liquids (such as coffee, black or green tea, red bull, chocolate) after midnight of the evening before the study session, as well as during the study day.
7. Participants must be willing not to drive a traffic vehicle within 48 h following MDMA administration.
8. Women of childbearing potential must have a negative pregnancy test at the beginning of the study and must agree to use an effective form of birth control. Pregnancy tests are repeated before each study session.
9. Body mass index: 18-27 kg/m^2

10.4.3 Exclusion criteria

1. Chronic or acute medical condition including clinically relevant abnormality in physical exam, laboratory values, or ECG. In particular: Hypertension (>140/90 mmHg) or Hypotension (SBP<85 mmHg). Personal or first-grade history of seizures. Cardiac or neurological disorder.
2. Current or previous psychotic or major affective disorder
3. Psychotic or major affective disorder in first-degree relatives
4. Prior illicit drug use (except THC-containing products) more than 5 times or any time within the previous 2 months.
5. Pregnant or nursing women.
6. Participation in another clinical trial (currently or within the last 30 days)
7. Use of medications that are contraindicated or otherwise interfere with the effects of the study medications (monoamine oxidase inhibitors, antidepressants, sedatives etc.)
8. Tobacco smoking

STUDY PROCEDURES

10.5 Schedule of Events

The schedule of events for a subject is shown on **table 1**. Subjects take part in a 1-h screening session, four 10-h test sessions plus a next-day single blood sampling appointment (30 min), and a 1-h end of study (EOS) visit.

Table 1: Schedule of Events

Time	Week 0	Week 1	next day	Week 2	next day	Week 3	next day	Week 4	next day	
Event	screening	Session 1		Session 2		Session 3		Session 4		EOS
Duration (h)	2	7	1	7	1	7	1	7	1	1
Informed consent	x									
Interview	x									x
Medical exam	x									x
BP, HR	x	x		x		x		x		x
ECG	x									
Drug screen	x	(x)		(x)		(x)		(x)		
Pregnancy test	(x)	(x)		(x)		(x)		(x)		
Blood tests	x									x
CYP2D6 phenotyp	x									
PK blood samples		x	x	x	x	x	x	x	x	
Adverse events		x	x	x	x	x	x	x	x	x

BP=blood pressure, HR=heart rate, ECG=electrocardiogram, CYP=cytochrome P450 enzyme, PK=pharmacokinetik, EOS=end of study tests

10.6 Screening procedure

10.6.1 Informed consent

The subjects will be informed about the study both verbally and by the approved written consent form regarding MDMA and bupropion, the study procedures, and associated risks. The investigator and the subject will both personally sign and date the consent form as confirmation of consent.

10.6.2 Physical health

Subjects will be examined by a study physician. Basic health will be ensured by general medical examination including medical history, physical examination, ECG, and blood chemistry and hematology. Body weight will also be measured.

10.6.3 Mental health

Subjects will be screened using a semi-structured clinical interview for DSM-IV [48] to exclude those with a personal or family (first-degree relative) axis I major psychiatric disorder or a history of illicit drug dependence.

10.6.4 History of Drug use

Occasional recreational drug use in the past is not an exclusion criterion if no adverse reactions occurred and if use was moderate and controlled. Drugs screens are primarily intended to document and control for concomitant drug use and not as a means to exclude subjects from the study. However, subjects will be asked to abstain from any illicit drug use during the study and drug screens are performed during screening and randomly before test-sessions. Positive screens for stimulants, opioids or hallucinogens will result in exclusion from the study (next study session). Smokers will not be included in the study. Subjects will also be asked to abstain from excessive alcohol consumption between test-sessions and in particular to limit their use to one glass on the day before the test sessions. Positive screens for THC (cannabis) will be recorded but do not result in study exclusion. This is because THC consumption can be detected in urine for up to several weeks and use days before a study day is unlikely to affect the outcome. Also we expect few if any positive screens.

10.6.5 Screening laboratory tests

The following laboratory blood tests are performed at the screening examination: Na, K, creatinine, ASAT, ALAT, γ-GT, hemoglobin, hematocrit, white blood cell count, red blood cell count, platelet cell count. Urine drug screen (Triage-8, Biosite, Morges, CH). Urine pregnancy test.

10.6.6 CYP2D6 phenotyping

Subjects will be asked to ingest a tablet of dextromethorphan (Bexin®) 25 mg in the evening of the screening day or on a day prior to the test sessions (10-11PM) and to collect and measure the amount of urine during the next 8 h (night urine plus first morning urine).

10.6.7 Genotyping

Polymorphisms in gens that code for monoamine transporters and enzymes contribute to inter-individual variations in the clinical response to amphetamines and to stimulant dependence [49]. We will collect an EDTA 7.5 ml blood sample for genotyping of gens coding for the monoamine transporters (SLC6A2A and others) and metabolic enzymes (CYP2D6, catecholamine O-methyl-transferase). Subjects are asked to sign a separate inform consent form for genotyping. It is no study exclusion if a subject refuses the genotyping test.

10.6.8 Personality

Personality traits are known to affect subjective responses to amphetamines [50]. The NEO-five factor inventory (NEO-FFI)[51] and the Freiburger Personality Inventory [52] will be used to assess personality traits and their potential modulatory effects on the response to MDMA. In addition, the German version of the Interpersonal Reacitivity Index (IRI), the Saarbrücker Persönlichkeitsfragebogen (SPF) zur Messung von Empathie, will be used to assess trait empathy.

10.6.9 Menstrual cycle phase in women

Women exhibit differential subjective effects to amphetamines during the follicular as compared to the luteal phase of the menstrual cycle [53]. Therefore, female subjects will be tested during the follicular phase (day 2-14) of the cycle.

10.7 Schedule of experimental session

Table 2 shows the schedule of events for each experimental session. Each of the four test sessions lasts 10 h from 8 AM to 6 PM. Subjects will arrive at 8 AM at the Phase 1 Unit. Subjects will take the last dose of the bupropion (300 mg) or placebo pretreatment at the beginning of the study session at 8 AM. An indwelling intravenous catheter will be inserted into a subcutaneous vein of the forearm. MDMA (125 mg) or placebo is administered at 10 AM (2 h after bupropion/placebo). The acute effects of MDMA last for 4 h [3]. Outcome measures (see below) will repeatedly be assessed during the study session. The sessions end at 6 PM. Subjects will be under continuous medical control until the effects of the drug have completely subsided (within 4-6 h after MDMA administration).

Table 2: Schedule of events for each session

Time	08:00	09:00	10:00	10:20	10:40	11:00	11:15	11:30	12:00	12:30	13:00	14:00	15:00	16:00	18:00	10:00	blood samples
relative Time (min/h)	-120	-60	0	20	40	60	75	90	120	150	180	4	5	6	8	24	
Bupropion (300 mg)	X																
MDMA (125 mg)			X														
neuroendocrine samples																	
cortisol, prolactin	0								1								2
copeptin, oxytocin	0					1			2								3
catecholamines	0					1			2								3
PK blood sample	-1		0	1	2	3		4	5	6	7	8		9	10	11	13
Psychometrics																	
VAS	-2	-1	0	1	2	3		4	5	6	7	8	9	10	11	12	
AMRS	0						1		2				3			4	
ARCI	0						1			2							
LC	0												1			2	
5D-ASC													1				
AE	3										1					2	
Physiological measures																	
blood pressure	-2	-1	0	1	2	3		4	5	6	7	8	9	10	11		
heart rate	-2	-1	0	1	2	3		4	5	6	7	8	9	10	11		
body temperature	-2	-1	0	1	2	3		4	5	6	7	8	9	10	11		
pupil diameter	-2	-1	0	1	2	3		4	5	6	7	8	9	10	11		
Cognitive Tests																	
FERT1, MECT								1									
FERT2									1								
MET											1						
SVO												1					
PK=pharmacokinetic, AMRS=adjectiv mood rating scale, 5D-ASC=altered states of consciousness scale, VAS=visual analog scales, ARCI=adiction reserach inventory, LC=list of complaints, AE=adverse events, FERT=face emotional recognition taks, MECT=mixed-emotions choice task, MET, multifaceted empathy test, SVO, social value orientation																	21

10.8 Assessments and Measures

10.8.1 Psychometric assessments

10.8.1.1 Visual Analog Scales (VAS)

Previously tested and sensitive VAS will be repeatedly used to better characterize subjective drugs effects over time [3, 54]. VAS will be presented as 100 mm long horizontal lines marked with “not at all” on the left and “extremely” on the right [3]. The following VAS will be used: “drug effect”, “good effect”, “bad effect”, “liking”, “high”, “happiness”, “energy level”, “feelings of closeness to others”, “satisfied”, “mind racing”, “ability to concentrate”, “fear”, “hungry”, “sedated”, “talkative”, “open”, “want to be hugged”, “want to hug”, “be alone”, “be with others”, and “confused”. Scales will be administered 120 and 60 min before and 0, 20, 40, 60, 90, 120, 150, 180 min, and 4, 5, 6, 8, and 24 h after MDMA/placebo administration.

10.8.1.2 Adjective mood rating scale (AMRS)

The adjective mood rating scale (AMRS) is a 60-item Likert scale that allows repeated assessment of mood in 6 dimensions: activation, inactivation, well-being, anxiety/depressed mood, extro- and introversion, and emotional excitability. The scale is sensitive to the effects of MDMA [3]. It will be used before, 75, 120 min, 5 h, and 24 h after MDMA administration. The German EWL60S version of the AMRS is used [55].

10.8.1.3 Addiction Research Center Inventory (ARCI)

The Addiction Research Center Inventory (ARCI) is a true-false questionnaire with five empirically derived scales: pentobarbital-chlorpromazine-alcohol group, a measure of sedation; morphine-benzedrine group, a measure of euphoria; lysergic acid diethylamine group (LSD), a measure of dysphoria and somatic symptoms; benzedrine group, a stimulant scale consisting mainly of items relating to intellectual efficiency and energy; and amphetamine, an empirically derived scale sensitive to the effects of d-amphetamine [56]. It has previously been shown to be sensitive to the effects of MDMA [47, 57] and is available in a validated German version [58]. The ARCI will be administered before, at 75 min, and 150 min after MDMA administration.

10.8.1.4 Altered states of consciousness (5D-ASC)

The 5 dimensions of altered states of consciousness (5D-ASC) scale is a visual analog scale consisting of 94 items [59, 60]. The instrument is constructed of five scales and allows assessing mood, anxiety, derealization, depersonalization, changes in perception, auditory alterations, and

reduced vigilance. The scale is well-validated and widely used in psychopharmacological research [60]. The 5D-ASC scale will be administered once 5 h after MDMA administration to retrospectively assess drug peak effects.

10.8.2 Physiological assessments

10.8.2.1 Vital signs

Blood pressure, heart rate, body temperature, and pupil diameter will be recorded at -120, -60, 0, 20, 40, 60, 90, 120, 150, 180 min, and 4, 5, 6, and 8 h after MDMA/placebo administration. Blood pressure (systolic and diastolic) and heart rate will be measured using an automatic oscillometric device. Body temperature will be measured using an ear thermometer. Ambient temperature is also recorded three times on the experimental day. Pupil diameter and the reaction to a light stimulus will be recorded using a handheld dynamic pupillometer (PRL-200, Neuroptics, Irvine, CA, USA) [61].

10.8.2.2 List of complaints (LC) and Adverse effects (AE)

Adverse effects (AE) will be assessed systematically during the session using a modified list of complaints (LC) [62]. The scale consists of 66 items, yielding a global score measuring physical and general discomfort. The LC list is administered 1 h before and 5 h after MDMA/placebo. An additional LC is completed 24 h after MDMA/placebo. Additional AE during the session will be listed in the case report form (CRF) 6 h after MDMA/placebo administration. Subjects will also be asked to report AE between study sessions at the beginning of the next session.

10.8.2.3 Emotion recognition

Facial emotion recognition task (FERT1)
Stimuli consist of facial pictures from the Ekman and Friesen series [63]. The facial emotional recognition task (FERT1) is sensitive to serotonin or norepinephrine uptake inhibition and to MDMA [31, 64]. The version of the task employed includes four basic emotions (anger, fear, happiness, and sadness), with pictures morphed between neutral (0%) and prototype emotion (100%) in 10% increments [65]. For each emotion, four different actors (two of each gender) were employed, resulting in 40 stimuli for each of four emotions (10 incrementally morphed pictures/actor/emotion). In addition, 10 neutral stimuli were added, for a total of 170 pictures. Faces will be presented in randomized order for 500 msec and replaced by a rating screen. Participants rate each face by selecting the emotion depicted, from the four emotions and neutral. The main outcome measure is accuracy (proportion correct).

Mixed emotions forced-choice task (MECT)
This task is based on a paradigm previously described by [66] and set-up by Domes [67]. Slides of two male and two female actors were chosen from the NimStim set of facial expressions [68] and were morphed from one emotional expression to another in 10% steps, resulting in series of 5 pictures showing blends of two basic emotions: e.g. 70% anger/30% fear, 60% anger/40% fear, 50% anger/50% fear, 40% anger/60% fear, 30% anger/70%fear. The expressions morphed were anger to happiness, anger to sadness, anger to fear, happiness to sadness, and fear to sadness, and fear to happiness. The pictures are projected separately on a screen in randomized order. In a forced-choice decision task, participants are asked to choose as quickly as possible which particular emotion the face displayed. A corresponding button press stops the presentation and another face is displayed. Pictures are randomly presented within blocks of 20 faces of one particular blend of two emotions. In total, 6 blocks are presented (120 pictures). Button presses and response latencies are recorded.

Emotion recognition task (FERT2)
To examine sensitivity to facial affect in a naturalistic way, we will employ a procedure with neutral faces, which “developed” a specific emotion in a pseudo-continuous way [67, 69, 70]. For this task, slides of the six basic emotions (fear, sadness, disgust, happiness, anger, surprise) were used from the NimStim set of facial expressions [68]. Using a computer program (Winmorph 2.0, Softonic International S.L., Barcelona, Spain), they were electronically morphed by 1% steps of intensity from 0% (neutral) to 100% of intensity of a specific emotion. Three female and three male pictures were chosen, resulting in 36 sets of faces (6 emotions × 6 faces) with 100 pictures each. Each picture of a

particular set is presented for 80 milliseconds, beginning with 0% intensity and increasing up to 100% in 1% steps. Participants are instructed to press a stop button when they feel subjectively certain of becoming “aware of the emotion the face is beginning to show.” This instruction is intended to reduce false alarms. After stopping the presentation, a forced choice between the six basic emotions is asked for. As dependent variables, we record the emotion intensity at which the presentation was stopped and the emotion category chosen after stopping. The 36 trials of one block are presented in a randomized order and are presented twice, resulting in 72 trials.

10.8.2.4 Empathy and social value orientation

We will characterize “empathogenic” and social effects of MDMA in collaboration with Dr. Christoph Eisenegger, University of Cambridge, UK, and Prof. Boris Quednow, University of Zurich. The tests are used by our collaborators in similar settings and are entertaining for the subjects, thus neither very demanding nor stressful. The tests will be performed towards the end of the test day when subjects are generally relaxed and still experience an attenuated drug effect.

Multifaceted Empathy Test (MET)
The Multifaceted Empathy Test (MET) [71] is a PC-assisted test consisting of photographs that show 23 pairs of picture stimuli with people in emotionally charged situations. The MET is a naturalistic measure of empathy that allows separate assessments of cognitive and emotional aspects of empathic functioning. To assess cognitive empathy, participants are required to infer the mental state of the subject in the photo, and are asked to indicate the correct one from a list of four. Then, to assess emotional empathy, subjects rate their emotional reactions in response to the pictures (emotional empathy). Participants indicate their responses on a visual analogue scale ranging from 0 to 9 (0=not at all, 9=very much). The test requires about 15 min and is performed 3 h after MDMA/placebo administration.

Social Value Orientation (SVO) task
We will use a Social Value Orientation (SVO) task [72, 73] to address effects of MDMA in the self/other allocation plane. We hypothesize that MDMA produces a shift from a more competitive/individualistic to a more prosocial/altruistic orientation/state. We will use the recently developed validated and sensitive 15-item paper version of the SVO slider measure [74]. The test requires about 10 minutes and is performed 4 h after MDMA/placebo administration.

10.8.2.5 End of Session Questionnarie

At the end of each session, participants will be asked to indicate how much they liked the drug overall and how strong the overall effect was on separate VAS [57].

10.8.3 Neuroendocrine function

MDMA increases cortisol, prolactin, catecholamine, and 5-HT concentrations in plasma in humans [75-78]. We will test the effect of bupropion on MDMA-induced increases in plasma cortisol, prolactin, catecholamines, testosterone, as well as on progesterone and estradiol levels (only women). Catecholamine samples will be collected at -120, 60, and 120 min after MDMA/placebo administration (3x7.5 ml blood). Plasma cortisol, prolactine, testosterone, progesterone, and estradiol samples will be collected 120 min before and 120 min after MDMA/placebo administration (2x7.5 ml blood). MDMA also increases vasopressin (AVP) and oxytocin and both may modulate some of the effects of MDMA [79, 80]. Copeptin is released stoichiometrically together with AVP and is easier to measure than AVP. Copeptin and oxytocin samples will be collected at -120 min, 60 min, and 120 min after MDMA/placebo administration (3x7.5 ml blood).

10.8.4 Blood sample collection (pharmacokinetics)

Blood samples for the determination of drug concentrations over time (13x7.5 ml) will be collected on the study day -2, 0, 0.33, 0.67, 1, 1.5, 2, 2.5, 3, 4, 6, and 24 h after MDMA or placebo administration. This will allow us to assess the pharmacokinetics of both MDMA and bupropion and potential PK interactions of the two compounds. Blood samples will be collected in heparinized tubes, centrifuged at 4°C at 2000 rpm for 10 min, and the plasma is then stored at -20°C (two aliquots) until analysis. Collection tubes labels will include the following information: study number, study day, subject number,

and sample number. The date and actual blood sampling time will be recorded on the sample collection page in the CRF.

10.8.4.1 Amount of blood samples

On each of the four study days a total of 21 blood samples (21x7.5 ml = 157.5 ml) will be collected amounting to a total of 630 ml of blood during the whole study. This amounts to about 25% more than a standard blood donation. However, blood is collected in amounts of 157.5 ml every 2 weeks spread out over 6 weeks and not at once which allows for regeneration. In order to blind the study, plasma samples will be collected also on the placebo day.

10.8.5 End of Study (EOS) visit

10.8.5.1 EOS Examination

Clinical examination and vital signs. Blood tests: Na, K, creatinine, ASAT, ALAT, γ-GT, hemoglobin, hematocrit, white blood cell count, red blood cell count, platelet cell count. Adverse effects.

10.8.5.2 EOS Questionnaire

A debriefing interview will be performed including a retrospective comparative evaluation of the subjective experience of all four study sessions. Subjects will be asked to rate the overall drug effect for each of the four sessions. Subjects will also be asked to guess the assignment of the study drugs to the four study sessions to assess whether subjects were able to distinguish the study conditions (bupropion vs. placebo). They will also be asked to provide a feed-back on the study and their experience and to indicate whether they would take the drug again in a controlled or recreational setting [1].

10.9 Study Drugs

10.9.1 Bupropion

Bupropion is a dopamine and norepinephrine transporter inhibitor used in the treatment of depression and nicotine dependence [8, 46]. **Dose selection:** Bupropion is clinically used in doses of 150-300 mg once daily (Wellbutrin XR or Zyban). Experimental studies in healthy subjects have used single doses of 150 mg [81], 150 mg once-daily for 7 days [82] or 150 mg twice-daily (300 mg) for 6 days [10]. The latter dosing regimen reduced subjective effects of methamphetamine in humans. In healthy subjects, treatment with bupropion 150 mg SR once-daily for 3 days followed by 150 mg twice-daily for 8 days produced a 26% DAT occupancy as measured by ^{11}C-βCIT-FE PET 3 hours after the last dose of bupropion [16]. In contrast, no significant striatal DAT occupancy was observed 2.5 h following administration of a single 150 mg as measured by 11C-raclopride PET in humans [83]. Based on these data we will use a chronic 7-day dosing regimen with uptitration. **Dose:** Bupropion will be administered in a dose of 150 mg (Wellbutrin XR) once-daily in the morning for three days followed by 300 mg (2x150mg) for 4 days before the test day. The same regimen is used to initiate treatement with bupropion for smoking cessation [46]. On the test day, a final 300 mg dose will be administered at 8:00 AM, 2h before the administration of MDMA at 10:00 AM. **Pharmacokinetics:** T_{max} (Wellbutrin XR): 5h, [46]. $T_{1/2}$: 20h [46]. Bupropion is an inhibitor of the CYP 2D6 enzyme [46]. CYP 2D6 metabolizes MDMA. Because MDMA inhibits its own metabolism by mechanism-based CYP 2D6 inhibition the additional effect of bupropion is expected to result in a minimal increase in the exposure of MDMA by maximally 20% as previously documented for the CYP2D6 inhibitor paroxetine [47]. **Tolerability:** Bupropion at doses of 300 mg is well tolerated in humans. Expected frequent (<1/10) adverse effects include urticaria, anorexia, tremor, dizziness, and increased blood pressure. Expected very frequent (>1/10) effects are: insomnia (30%), headache (13%), dry mouth (12%), and nausea (10%). Severe but rare (<1/1'000) events include hypersensitivity reactions and seizures [46]. **Drug preparation:** Bupropion (Wellbutrin XR® 150 mg, GlaxoSmithKline) will be purchased as the original marketed product (no contract with the company). Tablets will be encapsuled with opaque gelatine capsules for blinding purposes and similar placebo capsules containing manitol will be prepared. The lot number will be registered.

10.9.2 MDMA

10.9.2.1 Chemistry, manufacturing, control

Pharmaceutically pure racemic MDMA HCl (±3,4-methylenedioxymethamphetamine hydrochloride) (Lipomed AG, Arlesheim, Switzerland) will be used as in previous studies [3, 4]. MDMA has been provided by Prof. R. Brenneisen, Department for Clinical Research, University of Bern, Switzerland with the authorization of the Federal Office of Public Health (BAG), Bern. The investigator paid for the product (no contract). The drug was synthesized by Lipomed AG in 1998 (batch Nr. 94.1B5.51) and has a purity of 99.66%. Quality control of the drug confirmed purity (>99%) and content of the batch and the absence of decomposition products (Prof. Brenneisen, 30.1.2006, 23.7.2008, 12.1.2009 and 2.2011). MDMA from this lot has previously been used in human studies at the University Hospital of Psychiatry, Zurich, and is being used by us (EKBB 373/08; EKBB 253/09, EKBB 353/09), and by others [24, 84]. MDMA has been prepared as capsules (100 mg and 25 mg) by the Bichsel Laboratories, Interlaken. Identically looking placebo capsules contain manitol.

10.9.2.2 Metabolism and Pharmacokinetics of MDMA

After a 125 mg single dose of MDMA t_{max} is 2.5 h and $T_{1/2}$ is 9.6 h [3, 61]. MDMA inhibits its own metabolism possibly by forming an enzyme-metabolite complex with CYP2D6. This autoinhibition also explains the suggested non-linear pharmacokinetics of MDMA [85-88]. There are two main metabolic pathways for the metabolism of MDMA [87]. MDMA is N-demethylated to 3,4-methlyenedioxy-amphetamine (MDA). MDA is an active, but minor metabolite. CYP2D6-mediated O-demethylenation followed by COMT-catalyzed methylation leads to 4-hydroxy-3-mehoxymethamphetamine (HMMA) or 4-hydroxy-3-methoxyamphetamine (HMA). HMMA is the main metabolite of MDMA [87]. HMMA and HMA are rapidly glucuronidated or sulphate-conjugated. In this study we will determine the plasma-time profiles of MDMA, MDA, HMMA, and bupropion.

10.9.2.3 Dose selection (MDMA)

An oral dose of 125 mg MDMA corresponding to about 1.5-2.0 mg/kg will be used similar to previous studies [54, 61, 76, 88-91]. This dose of pure MDMA produces moderate subjective and cardiovascular effects [84, 90-92] and is considered safe in a controlled clinical setting [25, 88, 93, 94]. The same dose of 125 mg followed 2.5 h later by 62.5 mg MDMA has been used in clinical studies [23, 24]. Higher doses (150 mg or 2 mg/kg) have also been used without adverse events in MDMA studies [24, 91, 95, 96] and are used in clinical studies as well (150 mg plus 75 mg 2.5h later, P. Oehen, personal communication). Ecstasy tablets usually contain 80-160 mg of MDMA [97]. Use of more than one tablet at once is common.

10.9.3 Drug accountability, storage and return of study drugs

The study drugs are stored in a locked drug cabinet in a lockable room with restricted access at room temperature (15-25°C) at the Phase I Unit. A drug dispensing and accountability log for all capsules will be kept current. The log includes the date, origin, and amount of incoming study drug. The study physician/investigator dispensing the drug, registers and signs out the drug quantity, dispensing date of dispensing, participant number for all outgoing medications.

10.9.4 Concomitant medications

Concomitant use of medications will be recorded at the screening visit and at the beginning of each study session. Subjects are asked to abstain from using drugs that may interfere with the effects of the study medications including sleeping aids, cough medications, beta-blocker or other substances with potentially relevant psychoactive and cardiovascular effects. The PI may exclude subjects from study participation or reschedule sessions in the case of relevant pharmacokinetic of pharmacodynamic interactions with concomitant medications.

10.9.5 Randomization and blinding

Each subject will receive all treatments. Subjects and study personnel will be blinded to treatment order. Order is balanced and pseudo-random. A study subject number will be assigned to each subject. A treatment order is assigned to each subject number (code). Treatments are prepared and labeled according to the code by a pharmacist not involved in the study. The PI will also receive the code enclosed in an envelope. In case of a medical emergency the code can be accessed.

10.9.6 Compliance

The subjects will be reminded by a phone call or phone text message to ingest the bupropion/placebo pretreatment on the days before the test days. The medication containers will also be checked to confirm that the pretreatment was administered. On the test days medications are administered under observation by the study personnel. Finally, plasma samples for the determination of the concentration of bupropion will be collected on the test day also prior to the administration of the last dose of the pretreatment on the test day.

11 Analytics

11.1 MDMA and metabolites

Determination of plasma concentrations for MDMA and its metabolites MDA and HMMA as well as for bupropion will be performed in the Clinical Pharmacology laboratory in the Department of Biomedicine of the University Hospital Base using LC-MS/MS as previously reported [4].

11.2 CYP2D6 Phenotyping

Analysis of dextrorphan and dextromethorphan plasma levels is performed by LC-MS/MS by the Clinical Chemistry Department of the University Hospital of Basel.

11.3 Cortisol, prolactine, progesterone, estradiol, testosteron

Plasma hormone concentrations will be determined by routine standard immunoassays in the Clinical Chemistry Department of the University Hospital of Basel.

11.4 Catecholamines and metanephrines

Plasma catecholamines will be measured by HPLC with electrochemical detection by Dr. Eric Grouzmann, University Hospital Lausanne as previously reported [3].

11.5 Copeptin

Plasma copeptin levels will be measured with a sandwich immunoassay (BRAHMS AG, Hennigsdorf, Berlin, Germany) [98] in the Psychopharmacology laboratory group, Department of Biomedicine, University Hospital Basel as previously reported [80].

11.6 Oxytocin

Oxytocin levels will be analyzed by Prof. Inga Neumann, Department of Behavioral and Molecular Endocrinology, University of Regensburg, Germany using a radioimmunoassay [99].

12 Data analysis

12.1 Sample size estimation

Power analysis was performed with PASS®, Hintze J. Kaysville, Utah, US. For the primary outcome, which is the positive mood change, we consider a relative reduction of 20% in MDMA-induced “good drug effects” by bupropion to be clinically meaningful [3, 4, 47, 57]. A sample size of 13 achieves 82% power to detect a difference of 20 between the null hypothesis mean of 100 and the alternative hypothesis mean of 80 with a known standard deviation of 25 [100, 101] and with a significance level (alpha) of 0.05 using a two-sided one-sample t-test. While 13 subjects would be enough for meaningful results we will include 16 subject to have sufficient power, to be able to include equal numbers of both genders, and to allow for a balanced treatment order. Based on our experience with similar studies we assume a drop-out rate before inclusion of <33% and we will have to screen 24 subjects to include 16 in the study. Subjects who prematurely discontinue the study for any reason will be replaced if the number of discontinued subjects exceeds three. A samples size of 8 or less is commonly used in PK studies. A larger sample is used here to produce meaningful results for the pharmacodynamic parameters.

12.2 Analysis of outcomes

Psychometric measures will be analyzed by the PI using standardized and published procedures [3, 4]. E_{max} and AUEC values will be determined with repeated measurements. Values will then be compared using two-way factorial ANOVA, with the factors MDMA (MDMA vs. placebo) and bupropion (bupropion vs. placebo) followed by Tukey post hoc comparisons. Transformations will be made before analysis, if response variables are non-normally distributed. Pharmacokinetic data will be analyzed using non-

compartmental methods. Statistical analyses will be performed using Prism 5® (GraphPad, La Jolla, CA) and Statistica® (StatSoft, Tulsa, OK). C_{max} and t_{max} will be obtained directly from the observed data and AUC and $t_{1/2}$ values determined by PK Functions for Excel®. AUC values will be calculated by the trapezoidal rule.

13 Protection of subjects

Trained study personnel will place venous accesses, administer the study drugs and collect blood samples. Subjects will be supervised by trained study personnel during the entire test sessions, backed-up by study physicians. After the test session subjects are allowed to leave only if the subjective and cardiovascular effects of MDMA have completely ceased (see specific toxicity of MDMA and monitoring). In the case of unexpected prolonged or severe adverse reactions subjects will remain in the hospital and are monitored as long as necessary. Subjects will be provided with the phone number of the study physician in case of an emergency between/after study sessions.

13.1 Potential risks

Physical risks:
MDMA: A large series of experimental laboratory studies have been performed using MDMA in healthy subjects [1, 3, 4, 6, 7, 42, 54, 57, 61, 75, 76, 84, 87-92, 95, 96, 100-114]. The data indicates that the likelihood for significant toxicity from doses of MDMA such as the one to be used in the present study and in controlled settings is very low. Thus, no lasting biological or psychological injury is expected. At the University Hospital of Basel we have used MDMA alone or in the combination with other medications in approximately 100 healthy subjects in 200 experimental sessions. There were no severe or serious adverse effects. See below also for specific MDMA toxicity and monitoring.
Expected adverse effects of MDMA: MDMA is expected to produce moderate acute adverse effects such as increased jaw muscle tension, dry mouth, dizziness, palpitations, tremor, nausea, headache, insomnia, and transient anxiety in more than 10% of subjects, [1, 91]. MDMA is expected to produce sequelae (1-3 days post MDMA) including difficulty concentrating, irritability, or slightly depressed mood in more than 10% of subjects [1]. No severe or serious adverse effects are expected.
Bupropion: Bupropion is used to aid smoking cessation.
Expected adverse effects of bupropion: Bupropion at doses of 300 mg is well tolerated in humans. Expected frequent (<1/10) adverse effects include urticaria, anorexia, tremor, dizziness, and increased blood pressure. Expected very frequent (>1/10) effects are: insomnia (30%), headache (13%), dry mouth (12%), and nausea (10%). Severe but rare (<1/1'000) events include hypersensitivity reactions and seizures [46].
Venipunction: There is a risk for pain, bruising and thrombophlebitis.
Risk to privacy of subjects: Potential risks of data collection include breach of confidentiality. Financial risks: There is no risk of expense to the subject. Insurance coverage is provided. Travel expenses are expected to be minimal and are included in the compensation of 1'000 CHF per subject.

13.1.1 Specific toxicity of MDMA and monitoring

Duration of monitoring: Subjective and cardiovascular effects of MDMA last only for a mean of 3.5 h and completely subside within 5-6 h [76, 77, 84, 91, 101]. Subjects are monitored for 8 h after MDMA administration because PK samples are taken up to this time and to account for any unforeseen prolonged MDMA effects due to the administration of bupropion. In our previous studies, that similarly evaluated interactive effects of MDMA with various pretreatments, subjects were dismissed 4 h after MDMA administration [1, 42, 100, 101]. Another interaction study used an observation time of 6 h after MDMA administration shorter than the present study [77]. Only one or two subjects are tested simultaneously on the same test day.
Cardiovascular effects: Blood pressure and heart rate are measured repeatedly (Table 2). Closer monitoring will be implemented if blood pressure values exceed 180/120 mm Hg or decrease 90 mm Hg für SBP. Subjects will be monitored for signs of hypertensive crisis such as headache, angina pectoris or neurological deficits. Subjects will remain at the Phase 1 Unit overnight for further observation if blood pressure is >140/95 mmHg or <90/60 mmHg or pulse rate is >100/min or <45/min at the end of the test session. Treatment of a hypertensive reaction (P_{sys}>220 mmHg) would include administration of oxygen, lorazepam, nitroglycerin or nifedipine. In the case of angina pectoris, oxygen is given and ECG monitoring is initiated and cardiac enzymes will be measured. Treatment of hypotension would include trendelenburg position, infusion of 500 ml of saline, and ephedrine or norepinephrine as needed.

<u>Psychological effects:</u> The effects of MDMA are mostly pleasurable. Dysphoric reactions to MDMA and moderate anxiety may occur at the onset of the MDMA effect. Such effects have always resolved within hours [91]. Unexpected severe anxiety would be treated with a benzodiazepine. Up to one third of the subjects are expected to experience slightly depressed mood including emotional irritability, lack of energy, brooding and bad dreams for up to 3 days following MDMA administration [91]. These effects will be specifically monitored using an adverse effects check list. After the test session subjects are allowed to leave only if the subjective effects of MDMA have completely ceased (VAS score "drug effect" back to pre-dose levels ± 10%).
<u>Hyperthermia:</u> Increases in body temperature of approximately 0.5°C are expected [91]. If body temperature rises by more than 1.5°C appropriate treatment including hydration and cooling will be initiated [29]. Subjects will remain at the Phase 1 Unit overnight for further observation if body temperature has not returned to pretreatment levels ± 0.5 °C at the end of the test session.
<u>Dehydration/Hyperhydration:</u> Ecstasy use has been associated with both dehydration and hyperhydration leading to hyponatremia [29]. Subjects will be encouraged to drink no less than 1 but no more than 3 l during one session. If symptoms of hyponatremia such as confusion or vomiting are present, subjects will be further examined and serum sodium levels checked.
<u>Hepatotoxicity:</u> Ecstasy use has been associated with rare idiosyncratic liver toxicity [29]. Liver enzymes will be measured at the beginning and end of the study and any pathological results will be followed up.
<u>Neurotoxicity:</u> Recreational use of ecstasy has been linked to reversible alterations within the serotonin system ("neurotoxicity") [115, 116], especially when the drug is taken frequently and in high doses [30, 115]. The markers assessed in these studies likely represent functional neuronal changes and not damage to the neurons [117]. However, it is possible that high and repeated doses of ecstasy produce neurotoxicity. Several studies document no lasting effects of ecstasy in moderate ecstasy users [115, 118-120]. Even the use of ecstasy as recreational drug is currently considered to be associated with low harm compared to most other drugs [121]. There is no indication that administration of MDMA in a controlled setting has any adverse effects on cognitive function [25, 110, 122]. The use of pure MDMA in controlled settings is considered safe with regard to neurotoxic effects [93, 94]. No changes in serotonin transporter density were seen using [11C]-McN5652 PET four weeks after MDMA administration (1.5-1.7 mg/kg) in MDMA-naïve human volunteers [123, 124]. We will administer only two single moderate doses of MDMA similar to previous clinical studies [3, 23, 42, 47, 57, 80, 84, 90, 100, 101, 113, 114].
<u>Abuse liability:</u> The likelihood for future drug abuse triggered by the administration of MDMA in a controlled setting is assumed to be very low. MDMA has rewarding effects in animals and humans [95] but the risk for developing dependence is considered low when compared with other drugs of abuse [125]. In previous studies using MDMA in controlled settings, none of the participants expressed any interest in taking MDMA as a recreational drug after completing the study [1, 91]. Subjects with known risk factors for future MDMA use including depression or anxiety disorders [126] and prior drug dependence or other relevant illicit drug use [127] will be excluded from the study. Illicit drug use will also be monitored during the study using repeated urine drug screens.

13.2 Risk-Benefit Assessment

<u>Potential benefits:</u> First, the present study will generate objective, scientific information about the effects of MDMA that could not be obtained with observational studies. Second, research into the mechanisms of action of MDMA, which has strong mood-enhancing effects, will provide insight into the physiology of mood and pathophysiological processes involved in mood disorders. Third, normative data on the effects of pure MDMA in normal subjects in a controlled setting are useful to interpret results from studies in Ecstasy users. Forth, pharmacodynamics and pharmacokinetics of MDMA should be better characterized in the light of ongoing clinical phase II trials using MDMA. Fifth, we need to understand the mechanism of action of MDMA to treat intoxications with stimulants including Ecstasy. Finally, the study contributes to the efforts of finding medications for the treatment of stimulant dependence.

<u>Risk/benefit Ratio:</u> The risk for severe adverse effects or lasting physical harm is considered very low. With regard to subjective distress, the overall effect of MDMA is clearly a pleasurable experience with comparatively minimal risks for dysphoric reactions and tolerable adverse effects. The potential risk of further abuse of MDMA is expected to be very low in healthy subjects with low risk of drug abuse liability such as previous significant drug use or preexisting psychiatric disorder. The adverse effects of the bupropion pretreatment are also tolerable. Taken together, the risk and discomfort are minimal. Because healthy volunteers will participate in this phase I study, the benefits mainly consist in increased information that will not directly benefit the study participants but will be of benefit for the

society and patients with Ecstasy intoxications. However, based on our experience with similar studies, subject mostly experienced pleasure and considered the use of MDMA in a controlled setting to be of some interesting and personal value.

13.3 Monitoring of toxicity

13.3.1 Safety definitions

Adverse Event (AE):
Any untoward medical occurrence in a clinical trial subject administered an investigational medicinal product (IMP) and which does not necessarily have a causal relationship with this treatment. An AE can therefore be any unfavorable and unintended sign (including an abnormal laboratory finding), symptom, or disease temporally associated with the use of an (IMP), whether or not considered related to the IMP.

Adverse Reaction (AR):
All untoward and unintended responses to an IMP judged by investigator/sponsor as having a reasonable causal relationship to the IMP. The expression reasonable causal relationship means to convey in general that there is evidence or argument to suggest a causal relationship.

Unexpected Adverse Reaction (UAR):
An AR, the nature or severity of which is not consistent with the applicable product information (e.g. investigator's brochure for an unapproved investigational product or summary of product characteristics (SmPC) for an authorized product). When the outcome of the adverse reaction is not consistent with the applicable product information this adverse reaction should be considered as unexpected. Side effects documented in the IB or SmPC which occur in a more severe form than anticipated are also considered as being unexpected.

Serious Adverse Event (SAE) or Serious Adverse Reaction:
Any untoward medical occurrence or effect that at any dose results in death, is life-threatening, requires hospitalization, or prolongation of existing hospitalization, results in persistent or significant disability or incapacity or is a congenital anomaly or birth defect. In this context, the term life threatening refers to an event in which the trial participant was at immediate risk of death at the time of the event; it does not refer to an event, which might have caused death if it were more severe.

Suspected Unexpected Serious Adverse Reaction (SUSAR):
Any suspected adverse reaction related to an IMP that is both unexpected and serious.

Causality:
Most adverse events and adverse reactions that occur in this study, whether they are serious or not, will be expected treatment-related toxicities due to the medication used in this study. The assignment of the causality will be made by the investigator using the definitions in the table below.

Relationship	Description
**Unrelated**	There is no evidence of any causal relationship.
**Unlikely**	There is little evidence to suggest there is a causal relationship (e.g. the event did not occur within a reasonable time after administration of the IMP). There is another reasonable explanation for the event (e.g. the participant's clinical condition, other concomitant treatment).
**Possible**	There is some evidence to suggest a causal relationship (e.g. because the event occurs within a reasonable time after administration of the IMP). However, the influence of other factors may have contributed to the event (e.g. the participant's clinical condition, other concomitant treatments).
**Probable**	There is evidence to suggest a causal relationship and the influence of other factors is unlikely.

**Definitely**	There is clear evidence to suggest a causal relationship and other possible contributing factors can be ruled out.
**Not assessable**	There is insufficient or incomplete evidence to make a clinical judgment of the causal relationship.

13.3.2 Documentation

All adverse events occurring after the subject has signed the informed consent will be fully recorded in the subject's CRF. Each event will be described in detail along with start and stop dates, severity, relationship to investigational product, action taken and outcome.

13.3.3 Adverse events (AE) documentation

AE events will be described and recorded on the subject's CRF, regardless of the severity or relationship to the drug. Adverse effects are assessed by the standardized list of complaints (LC) [62]. Additional AEs are entered in the CRF at the end of the study session. AEs are also assessed 24 h after MDMA/placebo using the LC. Further, AEs are assessed before the next session typically 2 weeks after MDMA/placebo. AEs are rated for severity and the potential relationship to the study medications will be evaluated by the investigator according standard criteria. Subjects with AEs will be treated appropriately. Abnormal laboratory values will be repeated until normal or until the abnormality can be explained and the subject's safety is not at risk. In cases of a medical emergency, treatment is available in house and includes the 24h-availability of the reanimation team and intensive care facilities.

13.3.4 SAE and SUSAR reporting

Serious adverse events (SAE) are not expected. Should a SAE occur the PI will be informed and relationship to the study drug will be assessed by the PI using the above definitions. Complete information concerning the SAE will be collected by the PI and documented on a standard SAE form. Any SUSAR will be reported to the IRB and Swissmedic by the PI according to guidelines and within 7 days. The PI acts as sponsor in this investigator-initiated study. Therefore no reporting to a sponsor is needed. An annual safety report will be sent to Swissmedic by the PI.

13.3.5 Medical follow-up of adverse events

The investigator will ensure the subject receives medical follow-up as necessary until the condition has stabilized or returned to normal state, even if the period of the trial is over.

13.4 Ethical standards

The study will be performed in accordance with the Declaration of Helsinki, International Conference on Harmonization (ICH), Topic E6 Guidelines for Good Clinical Practice (GCP), the "Verordnung über klinische Versuche mit Heilmitteln" (VKlin), the "Heilmittelgesetz" (HMG), and the Standard Operating Procedures of the Phase 1 Research Unit (PRU) and Clinical Trial Unit (CTU) of the University Hospital Basel.

13.4.1 Institutional Review Board

The PI will submit the study protocol, the informed consent form, and related material to an approved institutional review board (IRB), the Ethische Kommission beider Basel (EKBB).

13.4.2 Protocol amendments

Any protocol amendments will require formal approval of the ethics committee prior to their implementation.

13.4.3 Early study termination

The study may be ended early in the case of unexpected adverse events or other reasons based on the PIs decision and on criteria determined as outlined below.

- An interim analysis will be performed after 2 subjects have completed the study. If this analysis indicates a significant increase in measures that indicate increased risk to the participants the study will be terminated early. Measures included in the analysis are: heart rate, blood pressure, the total of adverse effects (list of complaints), and adverse events.

- In addition, the following findings when due to MDMA or bupropion administration will trigger early analysis of results and potential study termination:
  - Hypertension $>P_{sys}$ 200 mm Hg or P_{dia} 140 mm Hg
  - Hypotension $<P_{sys}$ 85 mm Hg
  - Tachykardia >120 beats/min
  - Bradykardia < 45 beats/min
  - Serotonin syndrome (cloni, hyperthermia, delir)
  - Epileptic seizures
  - Other unexpected severe adverse effects

The PI will inform the IRB and Swissmedic when the study ends within 90 days or 15 days if the study is terminated early. A final report is sent to both within six months of study termination by the PI.

13.4.4 Insurance

Subjects will be insured for this trial via the University Hospital (see insurance certificate). The PI will be responsible for the initiation of all necessary action in the case of harm to a subject.

13.4.5 Compensation

Subjects will receive a financial compensation of CHF 1000.- for the completed trial. Pro rata payments will be done for early discontinuation. There are no other compensations.

13.4.6 Premature withdrawal of subjects

Subjects have the right to withdraw from the study at any time. The investigator may withdraw subjects from the study in the event of adverse events, protocol violations, or other reasons. An end of study assessment is made at the time of the subject's withdrawal. If the reason for removal of a subject from the study is an adverse event or an abnormal laboratory test result, the event or test will also be recorded on the CRF.

13.4.7 Replacement policy

Subjects who withdraw from the study will be replaced if the number exceeds three subjects, to maintain at least 13 subjects. Replacement subject will follow the same treatment sequence as the withdrawn subject.

13.5 Legal authorizations

MDMA is a scheduled illegal substance in Switzerland (Anhang d der BetmV-Swissmedic). We will apply to the BAG for permission to use MDMA in the present study after the study has been assessed by the IRB and Swissmedic.

13.6 Trial registration

The study will be registered at www.clinicaltrials.gov.

13.7 Study documentation and record keeping

The investigator will maintain adequate records to enable the conduct of the study to be fully documented. Copies of protocols, CRFs, originals of test result reports, drug dispensing logs, correspondence, records of informed consent and other documents pertaining to the conduct of the study will be kept on file for 10 years in the phase 1 unit archive. Identification codes will be kept for at least two years after study completion. All forms should be typed or filled out using a blue or black ball-point pen, and must be legible. Errors should be crossed out but not obliterated, the correction inserted, and the change initialed and dated by the investigator or an authorized person. For each subject enrolled a CRF will be completed and signed by the investigator. This also applies to those subjects who fail to complete the study.

13.8 Quality control and quality assurance

13.8.1 Training of personnel and SOPs

The study is performed in accordance with ICH GCP E6 and the SOPs of the Phase 1 Unit and the study coordination center of the CTU of the University Hospital Basel. The study personnel have completed a clinical investigator course of the CTU of the University Hospital Basel and additional on-site training.

13.8.2 Monitoring

Our trials are monitored by the Clinical Trial Unit of the Study Coordination Center Basel.

13.8.3 Direct access to source data

The investigator will permit study related monitoring visits, audits, ethics committee reviews, and regulatory inspections, and provide direct access to all source data. Source data are all information, original records of clinical findings, observations, or other activities in a clinical trial necessary for the reconstruction and evaluation of the trial.

13.8.4 Inspection

Inspections by regulatory authorities during study or after study closure might be performed to ensure proper study conduct and data handling procedures according to ICH-GCP guidelines and regulatory requirements. Inspections may include verification of all source documents, check of CRFs and site files and a visual inspection of the study site.

13.8.5 Confidentiality

The investigator will assure that subjects' anonymity will be maintained. At study inclusion a subject number is assigned to each subject. On CRFs and plasma samples subjects will not be identified by their names, but by the subject number. Data analysis is also performed using anonymized data files. The investigator will keep a log of subjects' numbers, names and addresses. This log that links the personal information with the study number is destroyed after study completion.

14 Time plan

Start of the study:	1.10.2012
Study duration per subject:	7 – 10 weeks
Study completion:	30.9.2013
Final report:	31.12.2013

15 Publications

All results will be presented at a congress meeting and published in a peer-reviewed scientific journal. The PI will be senior and corresponding author on all publications.

16 Budget and Funding

The costs for this study will be approximately 100'000 CHF not including salaries. The costs will mainly be covered by a grant of the Swiss National Science Foundation (SNF). The researchers do not receive any payments for the conduct of this research besides from their salaries.

17 Certifications of principal investigator

The PI is a MD specialized in internal medicine and clinical pharmacology and toxicology. The PI is attending physician at the Division of Clinical Pharmacology and Toxicology at the Department of Internal Medicine of the University Hospital Basel. The PI is also the group leader of the Psychopharmacology Research Group at the Department of Biomedicine of the University Hospital and the University of Basel. The PI completed a GCP and study physician training in 2003/2004 (University of Zurich) and holds a MAS degree in Clinical Research (2005-2007) of the University of California, San Diego, CA).

18 Conflict of interest

None

19 Research Environment

19.1 Study site

The study will take place in the fully equipped eight-bed Phase 1 Unit of the University Hospital Basel, Switzerland. This controlled setting allows for close monitoring of adverse effects and know-how and staff for PK sampling is available.

20 Responsibilities of the sponsor-investigator

Principal Investigator

**Name:** PD Dr. med. Matthias Liechti<br>**Address:** Dept. of Clinical Pharmacology<br>University Hospital Basel	will assure, that the study will be performed according to the local requirements and according to GCP-guidelines

The principal investigator is responsible for all aspects of the study conduct and the compliance with the protocol, the ICH GCP E6 guidelines, Swiss regulations, and the Decleration of Helsinki.

21 References

[1] Liechti ME, Vollenweider FX. The serotonin uptake inhibitor citalopram reduces acute cardiovascular and vegetative effects of 3,4-methylenedioxymethamphetamine ('Ecstasy') in healthy volunteers. J Psychopharmacol 2000;14:269-74.

[2] Liechti ME, Vollenweider FX. Which neuroreceptors mediate the subjective effects of MDMA in humans? A summary of mechanistic studies. Hum Psychopharmacol 2001;16:589-98.

[3] Hysek CM, Simmler LD, Ineichen M, Grouzmann E, Hoener MC, Brenneisen R, et al. The norepinephrine transporter inhibitor reboxetine reduces stimulant effects of MDMA ("ecstasy") in humans. Clin Pharmacol Ther 2011;90:246-55.

[4] Hysek CM, Simmler LD, Nicola V, Vischer N, Donzelli M, Krähenbühl S, et al. Duloxetine inhibits effects of MDMA ("ecstasy") in vitro and in humans in a randomized placebo-controlled laboratory study. PLoS One 2012;7:e36476.

[5] Hysek CM, Simmler LD, Liechti ME. Sex-differences in the effects of MDMA (ecstasy) on plasma copeptin in healthy subjects. Eur Neuropsychopharmacol 2011;21:S577.

[6] Hysek CM, Fink AE, Simmler LD, Donzelli M, Grouzmann E, Liechti ME. a-Adrenergic receptors contribute to the acute effects of MDMA in humans. Submitted 2012.

[7] Hysek CM, Schmid Y, Rickli A, Simmler LD, Grouzmann E, Liechti ME. Carvedilol inhibits the cardiostimulant and thermogenic effects of MDMA in humans. Swiss Medical Forum 2012;12:110S.

[8] Stahl SM, Pradko JF, Haight BR, Modell JG, Rockett CB, Learned-Coughlin S. A Review of the Neuropharmacology of Bupropion, a Dual Norepinephrine and Dopamine Reuptake Inhibitor. Primary care companion to the Journal of clinical psychiatry 2004;6:159-66.

[9] Peck AW, Hamilton M. Psychopharmacology of bupropion in normal volunteers. The Journal of clinical psychiatry 1983;44:202-5.

[10] Newton TF, Roache JD, De La Garza R, 2nd, Fong T, Wallace CL, Li SH, et al. Bupropion reduces methamphetamine-induced subjective effects and cue-induced craving. Neuropsychopharmacology 2006;31:1537-44.

[11] Shoptaw S, Heinzerling KG, Rotheram-Fuller E, Steward T, Wang J, Swanson AN, et al. Randomized, placebo-controlled trial of bupropion for the treatment of methamphetamine dependence. Drug Alcohol Depend 2008;96:222-32.

[12] Brensilver M, Heinzerling KG, Swanson AN, Shoptaw SJ. A retrospective analysis of two randomized trials of bupropion for methamphetamine dependence: Suggested guidelines for treatment discontinuation/augmentation. Drug Alcohol Depend 2012.

[13] Elkashef AM, Rawson RA, Anderson AL, Li SH, Holmes T, Smith EV, et al. Bupropion for the treatment of methamphetamine dependence. Neuropsychopharmacology 2008;33:1162-70.

[14] McCann DJ, Li SH. A novel, nonbinary evaluation of success and failure reveals bupropion efficacy versus methamphetamine dependence: reanalysis of a multisite trial. CNS neuroscience & therapeutics 2012;18:414-8.

[15] Gruner JA, Marcy VR, Lin YG, Bozyczko-Coyne D, Marino MJ, Gasior M. The roles of dopamine transport inhibition and dopamine release facilitation in wake enhancement and rebound hypersomnolence induced by dopaminergic agents. Sleep 2009;32:1425-38.

[16] Learned-Coughlin SM, Bergstrom M, Savitcheva I, Ascher J, Schmith VD, Langstrom B. In vivo activity of bupropion at the human dopamine transporter as measured by positron emission tomography. Biological psychiatry 2003;54:800-5.

[17] Stoops WW, Lile JA, Glaser PE, Hays LR, Rush CR. Influence of acute bupropion pre-treatment on the effects of intranasal cocaine. Addiction 2012;107:1140-7.

[18] Oliveto A, McCance-Katz FE, Singha A, Petrakis I, Hameedi F, Kosten TR. Effects of cocaine prior to and during bupropion maintenance in cocaine-abusing volunteers. Drug Alcohol Depend 2001;63:155-67.

[19] Poling J, Oliveto A, Petry N, Sofuoglu M, Gonsai K, Gonzalez G, et al. Six-month trial of bupropion with contingency management for cocaine dependence in a methadone-maintained population. Arch Gen Psychiatry 2006;63:219-28.

[20] Margolin A, Kosten TR, Avants SK, Wilkins J, Ling W, Beckson M, et al. A multicenter trial of bupropion for cocaine dependence in methadone-maintained patients. Drug Alcohol Depend 1995;40:125-31.
[21] Shoptaw S, Heinzerling KG, Rotheram-Fuller E, Kao UH, Wang PC, Bholat MA, et al. Bupropion hydrochloride versus placebo, in combination with cognitive behavioral therapy, for the treatment of cocaine abuse/dependence. Journal of addictive diseases 2008;27:13-23.
[22] SFA. Schweizerische Fachstelle für Alkohol- und andere Drogenprobleme. Berechnungen auf Basis der Schweizerischen Gesundheitsbefragung 2007. Available from www.sfa-ispa.ch 2009.
[23] Mithoefer MC, Wagner MT, Mithoefer AT, Jerome I, Doblin R. The safety and efficacy of ±3,4-methylenedioxymethamphetamine-assisted psychotherapy in subjects with chronic, treatment-resistant posttraumatic stress disorder: the first randomized controlled pilot study. J Psychopharmacol 2010;25:439-52.
[24] Oehen P. Phase II Pilot Randomized Double-Blind Placebo-Controlled Study of 3,4-Methylenedioxymethamphetamine (MDMA)Assisted Psychotherapy in Posttraumatic Stress Disorder (PTSD)- Switzerland. www.clinicaltrials.gov. ClinicalTrials.gov Identifier: NCT00353938. 2007.
[25] Sessa B, Nutt DJ. MDMA, politics and medical research: have we thrown the baby out with the bathwater? J Psychopharmacol 2007;21:787-91.
[26] Johansen PO, Krebs TS. How could MDMA (ecstasy) help anxiety disorders? A neurobiological rationale. J Psychopharmacol 2009;23:389-91.
[27] Dumont GJ, Sweep FC, van der Steen R, Hermsen R, Donders AR, Touw DJ, et al. Increased oxytocin concentrations and prosocial feelings in humans after ecstasy (3,4-methylenedioxymethamphetamine) administration. Social neuroscience 2009;4:359-66.
[28] Liechti ME, Kunz I, Kupferschmidt H. Acute medical problems due to Ecstasy use. Case-series of emergency department visits. Swiss Med Wkly 2005;135:652-7.
[29] Liechti ME. "Ecstasy" (MDMA): pharmacology, toxicology, and treatment of acute intoxication. Dtsch Med Wochenschr 2003;128:1361-6.
[30] McCann UD, Szabo Z, Scheffel U, Dannals RF, Ricaurte GA. Positron emission tomographic evidence of toxic effect of MDMA ("Ecstasy") on brain serotonin neurons in human beings. Lancet 1998;352:1433-7.
[31] Bedi G, Hyman D, de Wit H. Is ecstasy an "empathogen"? Effects of ± 3,4-methylenedioxymethamphetamine on prosocial feelings and identification of emotional states in others. Biological psychiatry 2010;68:1134-40.
[32] Hermle L, Spitzer M, Borchardt D, Kovar KA, Gouzoulis E. Psychological effects of MDE in normal subjects: are entactogens a new class of psychoactive agents? Neuropsychopharmacology 1993;8:171-6.
[33] Winstock AR, Mitcheson LR, Deluca P, Davey Z, Corazza O, Schifano F. Mephedrone, new kid for the chop? Addiction 2011;106:154-61.
[34] Prosser JM, Nelson LS. The toxicology of bath salts: a review of synthetic cathinones. J Med Toxicol 2012;8:33-42.
[35] Simmler LD, Buser TA, Hoener MC, Liechti ME. Effects of mephedrone and other "legal high" cathinone-derivatives on monoamine transport. 2012:submitted.
[36] Rothman RB, Baumann MH. Therapeutic and adverse actions of serotonin transporter substrates. Pharmacology & therapeutics 2002;95:73-88.
[37] Rothman RB, Baumann MH, Dersch CM, Romero DV, Rice KC, Carroll FI, et al. Amphetamine-type central nervous system stimulants release norepinephrine more potently than they release dopamine and serotonin. Synapse 2001;39:32-41.
[38] Gudelsky GA, Nash JF. Carrier-mediated release of serotonin by 3,4-methylenedioxymethamphetamine: implications for serotonin-dopamine interactions. Journal of neurochemistry 1996;66:243-9.
[39] Schindler CW, Gilman JP, Panlilio LV, McCann DJ, Goldberg SR. Comparison of the effects of methamphetamine, bupropion, and methylphenidate on the self-administration of methamphetamine by rhesus monkeys. Exp Clin Psychopharmacol 2011;19:1-10.
[40] Risbrough VB, Masten VL, Caldwell S, Paulus MP, Low MJ, Geyer MA. Differential contributions of dopamine D1, D2, and D3 receptors to MDMA-induced effects on locomotor behavior patterns in mice. Neuropsychopharmacology 2006;31:2349-58.
[41] Verrico CD, Lynch L, Fahey MA, Fryer AK, Miller GM, Madras BK. MDMA-induced impairment in primates: antagonism by a selective norepinephrine or serotonin, but not by a dopamine/norepinephrine transport inhibitor. J Psychopharmacol 2008;22:187-202.

[42] Liechti ME, Vollenweider FX. Acute psychological and physiological effects of MDMA ("Ecstasy") after haloperidol pretreatment in healthy humans. Eur Neuropsychopharmacol 2000;10:289-95.

[43] Brauer LH, de Wit H. Subjective responses to d-amphetamine alone and after pimozide pretreatment in normal, healthy volunteers. Biological psychiatry 1996;39:26-32.

[44] Brauer LH, De Wit H. High dose pimozide does not block amphetamine-induced euphoria in normal volunteers. Pharmacology, biochemistry, and behavior 1997;56:265-72.

[45] Tiihonen J, Kuoppasalmi K, Fohr J, Tuomola P, Kuikanmaki O, Vorma H, et al. A comparison of aripiprazole, methylphenidate, and placebo for amphetamine dependence. Am J Psychiatry 2007;164:160-2.

[46] AK. Arzneimittelkompendium der Schweiz. Basel: Documed AG, http://www.documed.ch, 2011.

[47] Farre M, Abanades S, Roset PN, Peiro AM, Torrens M, O'Mathuna B, et al. Pharmacological interaction between 3,4-methylenedioxymethamphetamine (ecstasy) and paroxetine: pharmacological effects and pharmacokinetics. J Pharmacol Exp Ther 2007;323:954-62.

[48] Wittchen HU, Wunderlich U, Gruschwitz S, Zaudig M. SKID-I: Strukturiertes Klinisches Interview für DSM-IV. Göttingen: Hogrefe-Verlag, 1997.

[49] Dlugos A, Freitag C, Hohoff C, McDonald J, Cook EH, Deckert J, et al. Norepinephrine transporter gene variation modulates acute response to D-amphetamine. Biological psychiatry 2007;61:1296-305.

[50] White TL, Lott DC, de Wit H. Personality and the subjective effects of acute amphetamine in healthy volunteers. Neuropsychopharmacology 2006;31:1064-74.

[51] Borkenau P, Ostendorf F, editors. NEO-Fünf-Faktoren-Inventar (NEO-FFI) nach Costa und McCrae. Göttingen: Hogrefe, 2008.

[52] Fahrenberg J, Hampel R, Selg H. Das Freiburger Persönlichkeitsinventar (FPI). Göttingen, Germany: Hogrefe, 1984.

[53] White TL, Justice AJ, de Wit H. Differential subjective effects of D-amphetamine by gender, hormone levels and menstrual cycle phase. Pharmacology, biochemistry, and behavior 2002;73:729-41.

[54] Kolbrich EA, Goodwin RS, Gorelick DA, Hayes RJ, Stein EA, Huestis MA. Plasma pharmacokinetics of 3,4-methylenedioxymethamphetamine after controlled oral administration to young adults. Therapeutic drug monitoring 2008;30:320-32.

[55] Janke W, Debus G. Die Eigenschaftswörterliste. Göttingen, Germany: Hogrefe, 1978.

[56] Martin WR, Sloan JW, Sapira JD, Jasinski DR. Physiologic, subjective, and behavioral effects of amphetamine, methamphetamine, ephedrine, phenmetrazine, and methylphenidate in man. Clin Pharmacol Ther 1971;12:245-58.

[57] Tancer M, Johanson CE. The effects of fluoxetine on the subjective and physiological effects of 3,4-methylenedioxymethamphetamine (MDMA) in humans. Psychopharmacology 2007;189:565-73.

[58] Bopp G, Bender W, Schütz CG. Validierung der deutschen Version des Addiction Research Center Inventory (ARCI). Suchtmedizin 2005;7:152-3.

[59] Dittrich A. The standardized psychometric assessment of altered states of consciousness (ASCs) in humans. Pharmacopsychiatry 1998;31 S2:80-4.

[60] Studerus E, Gamma A, Vollenweider FX. Psychometric evaluation of the altered states of consciousness rating scale (OAV). PLoS One 2010;5:e12412.

[61] Hysek CM, Liechti ME. Effects of MDMA alone and after pretreatement with reboxetine, duloxetine, clonidine, carvedilol, and doxazosin on pupillary light reflex. Psychopharmacology 2012;in press.

[62] Zerssen DV. Die Beschwerden-Liste. Münchener Informationssystem. München: Psychis, 1976.

[63] Ekman P, Friesen WV. Pictures of facial affect. Palo Alto: Consulting Psychologists, 1976.

[64] Harmer CJ, Shelley NC, Cowen PJ, Goodwin GM. Increased positive versus negative affective perception and memory in healthy volunteers following selective serotonin and norepinephrine reuptake inhibition. Am J Psychiatry 2004;161:1256-63.

[65] Young AW, Rowland D, Calder AJ, Etcoff NL, Seth A, Perrett DI. Facial expression megamix: tests of dimensional and category accounts of emotion recognition. Cognition 1997;63:271-313.

[66] Calder AJ, Young AW, Rowland D. Facial emotion recognition after bilateral amygdala damage: Differentially severe impairment of fear. Cognitive Neuropsychology 1996;13:699-745.

[67] Domes G, Czieschnek D, Weidler F, Berger C, Fast K, Herpertz SC. Recognition of facial affect in Borderline Personality Disorder. J Pers Disord 2008;22:135-47.
[68] Tottenham N, Tanaka JW, Leon AC, McCarry T, Nurse M, Hare TA, et al. The NimStim set of facial expressions: judgments from untrained research participants. Psychiatry Res 2009;168:242-9.
[69] Blair RJ, Colledge E, Murray L, Mitchell DG. A selective impairment in the processing of sad and fearful expressions in children with psychopathic tendencies. J Abnorm Child Psychol 2001;29:491-8.
[70] Coupland NJ, Singh AJ, Sustrik RA, Ting P, Blair R. Effects of diazepam on facial emotion recognition. J Psychiatry Neurosci 2003;28:452-63.
[71] Dziobek I, Rogers K, Fleck S, Bahnemann M, Heekeren HR, Wolf OT, et al. Dissociation of cognitive and emotional empathy in adults with Asperger syndrome using the Multifaceted Empathy Test (MET). J Autism Dev Disord 2008;38:464-73.
[72] Van Lange PAM. The pursuit of joint outcomes and equality in outcomes: An integrative model of social value orientation. J Pers Soc Psychol 1999;77:337-49.
[73] Haruno M, Frith CD. Activity in the amygdala elicited by unfair divisions predicts social value orientation. Nat Neurosci 2010;13:160-1.
[74] Murphy RO, Ackermann KA, Handgraaf MJJ. Measuring social value orientation Judgment and Decision 2011;in press.
[75] Grob CS, Poland RE, Chang L, Ernst T. Psychobiologic effects of 3,4-methylenedioxymethamphetamine in humans: methodological considerations and preliminary observations. Behavioural brain research 1996;73:103-7.
[76] Mas M, Farre M, de la Torre R, Roset PN, Ortuno J, Segura J, et al. Cardiovascular and neuroendocrine effects and pharmacokinetics of 3, 4-methylenedioxymethamphetamine in humans. J Pharmacol Exp Ther 1999;290:136-45.
[77] Dumont GJ, Kramers C, Sweep FC, Touw DJ, van Hasselt JG, de Kam M, et al. Cannabis coadministration potentiates the effects of "ecstasy" on heart rate and temperature in humans. Clin Pharmacol Ther 2009;86:160-6.
[78] Forsling M, Fallon JK, Kicman AT, Hutt AJ, Cowan DA, Henry JA. Arginine vasopressin release in response to the administration of 3,4-methylenedioxymethamphetamine ("ecstasy"): is metabolism a contributory factor? The Journal of pharmacy and pharmacology 2001;53:1357-63.
[79] Hysek CM, Domes G, Liechti ME. MDMA enhances "mind reading" of positive emotions and impairs "mind reading" of negative emotions. Psychopharmacology 2012;in press.
[80] Simmler LD, Hysek CM, Liechti ME. Sex differences in the effects of MDMA (ecstasy) on plasma copeptin in healthy subjects. The Journal of clinical endocrinology and metabolism 2011;96:2844-50.
[81] Carvalho AF, Kohler CA, Cruz EP, Sturmer PL, Reichman BP, Barea BM, et al. Acute treatment with the antidepressants bupropion and sertraline do not influence memory retrieval in man. European archives of psychiatry and clinical neuroscience 2006;256:320-5.
[82] Abler B, Gron G, Hartmann A, Metzger C, Walter M. Modulation of frontostriatal interaction aligns with reduced primary reward processing under serotonergic drugs. J Neurosci 2012;32:1329-35.
[83] Egerton A, Shotbolt JP, Stokes PR, Hirani E, Ahmad R, Lappin JM, et al. Acute effect of the anti-addiction drug bupropion on extracellular dopamine concentrations in the human striatum: an [11C]raclopride PET study. Neuroimage 2010;50:260-6.
[84] Kolbrich EA, Goodwin RS, Gorelick DA, Hayes RJ, Stein EA, Huestis MA. Physiological and subjective responses to controlled oral 3,4-methylenedioxymethamphetamine administration. J Clin Psychopharmacol 2008;28:432-40.
[85] O'Mathuna B, Farre M, Rostami-Hodjegan A, Yang J, Cuyas E, Torrens M, et al. The consequences of 3,4-methylenedioxymethamphetamine induced CYP2D6 inhibition in humans. J Clin Psychopharmacol 2008;28:523-9.
[86] Yang J, Jamei M, Heydari A, Yeo KR, de la Torre R, Farre M, et al. Implications of mechanism-based inhibition of CYP2D6 for the pharmacokinetics and toxicity of MDMA. J Psychopharmacol 2006;20:842-9.
[87] de la Torre R, Farre M, Roset PN, Pizarro N, Abanades S, Segura M, et al. Human pharmacology of MDMA: pharmacokinetics, metabolism, and disposition. Therapeutic drug monitoring 2004;26:137-44.
[88] de la Torre R, Farre M, Ortuno J, Mas M, Brenneisen R, Roset PN, et al. Non-linear pharmacokinetics of MDMA ('ecstasy') in humans. British journal of clinical pharmacology 2000;49:104-9.

[89] Cami J, Farre M, Mas M, Roset PN, Poudevida S, Mas A, et al. Human pharmacology of 3,4-methylenedioxymethamphetamine ("ecstasy"): psychomotor performance and subjective effects. J Clin Psychopharmacol 2000;20:455-66.
[90] Hasler F, Studerus E, Lindner K, Ludewig S, Vollenweider F. Investigation of serotonin-1A receptor function in the human psychopharmacology of MDMA. J Psychopharmacol 2009;23:923-35.
[91] Liechti ME, Gamma A, Vollenweider FX. Gender differences in the subjective effects of MDMA. Psychopharmacology 2001;154:161-8.
[92] Vollenweider FX, Gamma A, Liechti M, Huber T. Psychological and cardiovascular effects and short-term sequelae of MDMA ("ecstasy") in MDMA-naive healthy volunteers. Neuropsychopharmacology 1998;19:241-51.
[93] Vollenweider FX, Gamma A, Liechti M, Huber T. Is a single dose of MDMA harmless? Neuropsychopharmacology 1999;21:598-600.
[94] Lieberman JA, Aghajanian GK. Caveat emptor: researcher beware. Neuropsychopharmacology 1999;21:471-3.
[95] Tancer M, Johanson CE. Reinforcing, subjective, and physiological effects of MDMA in humans: a comparison with d-amphetamine and mCPP. Drug Alcohol Depend 2003;72:33-44.
[96] Tancer ME, Johanson CE. The subjective effects of MDMA and mCPP in moderate MDMA users. Drug Alcohol Depend 2001;65:97-101.
[97] Giroud C, Augsburger M, Sadeghipour F, Varesio E, Veuthey JL, Rivier L. [Ecstasy--the status in French-speaking Switzerland. Composition of seized drugs, analysis of biological specimens and short review of its pharmacological action and toxicity]. Praxis (Bern 1994) 1997;86:510-23.
[98] Morgenthaler NG, Struck J, Alonso C, Bergmann A. Assay for the measurement of copeptin, a stable peptide derived from the precursor of vasopressin. Clin Chem 2006;52:112-9.
[99] Landgraf R. Simultaneous measurement of arginine vasopressin and oxytocin in plasma and neurohypophyses by radioimmunoassay. Endokrinologie 1981;78:191-204.
[100] Liechti ME, Saur MR, Gamma A, Hell D, Vollenweider FX. Psychological and physiological effects of MDMA ("Ecstasy") after pretreatment with the 5-HT(2) antagonist ketanserin in healthy humans. Neuropsychopharmacology 2000;23:396-404.
[101] Liechti ME, Baumann C, Gamma A, Vollenweider FX. Acute psychological effects of 3,4-methylenedioxymethamphetamine (MDMA, "ecstasy") are attenuated by the serotonin uptake inhibitor citalopram. Neuropsychopharmacology 2000;22:513-21.
[102] Camarasa J, Marimon JM, Rodrigo T, Escubedo E, Pubill D. Memantine prevents the cognitive impairment induced by 3,4-methylenedioxymethamphetamine in rats. Eur J Pharmacol 2008.
[103] Kuypers KP, Ramaekers JG. Acute dose of MDMA (75 mg) impairs spatial memory for location but leaves contextual processing of visuospatial information unaffected. Psychopharmacology 2007;189:557-63.
[104] de la Torre R, Farre M, Mathuna BO, Roset PN, Pizarro N, Segura M, et al. MDMA (ecstasy) pharmacokinetics in a CYP2D6 poor metaboliser and in nine CYP2D6 extensive metabolisers. European journal of clinical pharmacology 2005;61:551-4.
[105] Farre M, de la Torre R, Mathuna BO, Roset PN, Peiro AM, Torrens M, et al. Repeated doses administration of MDMA in humans: pharmacological effects and pharmacokinetics. Psychopharmacology 2004;173:364-75.
[106] Pacifici R, Pichini S, Zuccaro P, Farre M, Segura M, Ortuno J, et al. Paroxetine inhibits acute effects of 3,4-methylenedioxymethamphetamine on the immune system in humans. J Pharmacol Exp Ther 2004;309:285-92.
[107] Vollenweider FX, Liechti ME, Gamma A, Greer G, Geyer M. Acute psychological and neurophysiological effects of MDMA in humans. J Psychoactive Drugs 2002;34:171-84.
[108] Hernandez-Lopez C, Farre M, Roset PN, Menoyo E, Pizarro N, Ortuno J, et al. 3,4-Methylenedioxymethamphetamine (ecstasy) and alcohol interactions in humans: psychomotor performance, subjective effects, and pharmacokinetics. J Pharmacol Exp Ther 2002;300:236-44.
[109] Liechti ME, Geyer MA, Hell D, Vollenweider FX. Effects of MDMA (ecstasy) on prepulse inhibition and habituation of startle in humans after pretreatment with citalopram, haloperidol, or ketanserin. Neuropsychopharmacology 2001;24:240-52.
[110] Liechti ME, Gamma A, Vollenweider FX. No lasting psycho-physiological effects of a single dose of MDMA ("Ecstasy") in controlled conditions in healthy human volunteers. Eur Neuropsychopharmacol 2001;11.
[111] Gamma A, Buck A, Berthold T, Liechti ME, Vollenweider FX. 3,4-Methylenedioxymethamphetamine (MDMA) modulates cortical and limbic brain activity as

measured by [H(2)(15)O]-PET in healthy humans. Neuropsychopharmacology 2000;23:388-95.
[112] Chang L, Grob CS, Ernst T, Itti L, Mishkin FS, Jose-Melchor R, et al. Effect of ecstasy [3,4-methylenedioxymethamphetamine (MDMA)] on cerebral blood flow: a co-registered SPECT and MRI study. Psychiatry Res 2000;98:15-28.
[113] Hysek CM, Brugger R, Simmler LD, Bruggisser M, Donzelli M, Grouzmann E, et al. Effects of the a_2-adrenergic agonist clonidine on the pharmacodynamics and pharmacokinetics of 3,4-methylenedioxymethamphetamine in healthy volunteers. J Pharmacol Exp Ther 2012;340:286-94.
[114] Hysek CM, Vollenweider FX, Liechti ME. Effects of a b-blocker on the cardiovascular response to MDMA (ecstasy). Emerg Med J 2010;27:586-9.
[115] Reneman L, Booij J, de Bruin K, Reitsma JB, de Wolff FA, Gunning WB, et al. Effects of dose, sex, and long-term abstention from use on toxic effects of MDMA (ecstasy) on brain serotonin neurons. Lancet 2001;358:1864-9.
[116] Buchert R, Thiele F, Thomasius R, Wilke F, Petersen K, Brenner W, et al. Ecstasy-induced reduction of the availability of the brain serotonin transporter as revealed by [11C](+)McN5652-PET and the multi-linear reference tissue model: loss of transporters or artifact of tracer kinetic modelling? J Psychopharmacol 2007;21:628-34.
[117] O'Callaghan JP, Miller DB. Neurotoxic effects of substituted amphetamines in rats and mice. In: Massaro EJ, editor. Handbook of Neurotoxicology Totowa: Humana Press Inc., 2010.
[118] de Win MM, Reneman L, Jager G, Vlieger EJ, Olabarriaga SD, Lavini C, et al. A prospective cohort study on sustained effects of low-dose ecstasy use on the brain in new ecstasy users. Neuropsychopharmacology 2007;32:458-70.
[119] de Win MM, Schilt T, Reneman L, Vervaeke H, Jager G, Dijkink S, et al. Ecstasy use and self-reported depression, impulsivity, and sensation seeking: a prospective cohort study. J Psychopharmacol 2006;20:226-35.
[120] Halpern JH, Pope HG, Jr., Sherwood AR, Barry S, Hudson JI, Yurgelun-Todd D. Residual neuropsychological effects of illicit 3,4-methylenedioxymethamphetamine (MDMA) in individuals with minimal exposure to other drugs. Drug Alcohol Depend 2004;75:135-47.
[121] Nutt DJ, King LA, Phillips LD. Drug harms in the UK: a multicriteria decision analysis. Lancet 2010;376:1558-65.
[122] Ludewig S, Ludewig K, Hasler F, Vollenweider FX. No lasting effects of moderate doses of MDMA (Ecstasy) on memory performance and mood states in healthy humans. Biological psychiatry 2003;53.
[123] Vollenweider FX, Gucker P, Schönbächler R, Kamber E, Vollenweider-Scherpenhuyzen MFI, Schubiger G, et al. Effects of MDMA on 5-HT uptake sites using PET and [11C]-McN5652 in humans. . Nervenarzt 2000;71.
[124] Vollenweider FX, Jones RT, Baggott MJ. Caveat emptor: editors beware. Neuropsychopharmacology 2001;24:461-3.
[125] Nutt D, King LA, Saulsbury W, Blakemore C. Development of a rational scale to assess the harm of drugs of potential misuse. Lancet 2007;369:1047-53.
[126] Huizink AC, Ferdinand RF, van der Ende J, Verhulst FC. Symptoms of anxiety and depression in childhood and use of MDMA: prospective, population based study. BMJ (Clinical research ed 2006;332:825-8.
[127] Pedersen W, Skrondal A. Ecstasy and new patterns of drug use: a normal population study. Addiction 1999;94:1695-706.
